# Antennal transcriptome analysis of odorant-binding proteins and characterization of GOBP2 in the variegated cutworm *Peridroma saucia*

**DOI:** 10.3389/fphys.2023.1241324

**Published:** 2023-08-10

**Authors:** Jun-Feng Dong, Ke Wang, Ya-Lan Sun, Cai-Hong Tian, Shao-Li Wang

**Affiliations:** ^1^ College of Horticulture and Plant Protection, Henan University of Science and Technology, Luoyang, China; ^2^ State Key Laboratory of Vegetable Biobreeding, Institute of Vegetables and Flowers, Chinese Academy of Agricultural Sciences, Beijing, China; ^3^ Institute of Plant Protection, Henan Academy of Agricultural Sciences, Zhengzhou, China

**Keywords:** antennal transcriptome, tissue expression, general odorant-binding protein, fluorescence binding assay, molecular docking

## Abstract

Odorant-binding proteins (OBPs) are expressed at extremely high concentrations in the chemo-sensilla lymph of insects and have long been thought to be crucial for delivering the semiochemicals to the odorant receptors. They are represented by multiple classes: general odorant-binding proteins (GOBP1 and GOBP2) and pheromone-binding proteins. In the current study, we identified a total of 35 OBPs in the antennal transcriptome of *Peridroma saucia*, a worldwide pest that causes serious damage to various crops. A gene expression value (TPM, transcripts per million) analysis revealed that seven OBPs (PsauPBP1/2/3, PsauGOBP1/2, PsauOBP6, and PsauOBP8) were highly abundant in the antennae. Next, we focused on the expression and functional characterization of PsauGOBP2. Real-time quantitative-PCR analysis demonstrated that *PsauGOBP2* was predominantly expressed in the antennae of both sexes. Fluorescence binding assays showed that the recombinant PsauGOBP2 strongly binds to the female sex pheromone components *Z*11-16: Ac (K_i_ = 4.2 μM) and *Z*9-14: Ac (K_i_ = 4.9 μM) and binds moderately (6 µM ≤ K_i_ ≤ 13 µM) to the host plant volatiles phenylethyl acetate, β-myrcene, and dodecanol. Further 3D structural modeling and molecular docking revealed that several crucial amino acid residues are involved in ligand binding. The results not only increase our understanding of the olfactory system of *P*. *saucia* but also provide insights into the function of PsauGOBP2 that has implications for developing sustainable approaches for *P*. *saucia* management.

## Introduction

Insects depend on olfaction system to locate oviposition sites, food sources, and mate partners, and to avoid natural enemies (Leal, 2013). The antennae are primary olfactory organs (Pelosi et al., 2006). Insect olfaction is orchestrated by the cooperation of multiple chemosensory proteins, mainly including chemosensory proteins (CSPs) (Pelosi et al., 2018; Li et al., 2021), odorant-binding proteins (OBPs) (Zhou, 2010; Pelosi et al., 2018), odorant-degrading enzymes (ODEs) (Vogt, 2003), odorant receptors (ORs) (Yang and Wang, 2000), sensory neuron membrane proteins (SNMPs) (Benton et al., 2007) and ionotropic receptors (IRs) (Benton et al., 2009).

Insect OBPs can bind and transport hydrophobic odorant molecules across the hydrophilic sensillum lymph to corresponding receptors on olfactory sensory neurons (Pelosi et al., 2018). Further, OBP-odorant complexes (or odorant itself) activate receptors (ORs or IRs) to stimulate a cascade of reaction, which converts chemical signals into electric signals and eventually lead to specific behaviors (Xu et al., 2005). Therefore, OBPs are essential for insects to recognize odorant molecules and can be utilized as targets for developing new behavioral disruptors/inhibitors (Zhou, 2010). The first insect OBP was characterized in *Antheraea polyphemus* in 1981 (Vogt and Riddiford, 1981). In the following 40 years, a plethora of OBPs have been identified in insects by molecular biology approaches especially omics techniques (Venthur and Zhou, 2018). Insect classic OBPs possess six conserved cysteines that constitute three disulfide bridges to form a binding cavity aligning some other amino acid residues (Leal et al., 1999; Lagarde et al., 2011). Meanwhile, OBPs with different numbers of conserved cysteines have also been found. These OBPs mainly include minus-C OBPs that have lost two conserved cysteines and plus-C OBPs with two additional conserved cysteines (Pelosi and Maida, 1995).

In Lepidoptera, general odorant-binding proteins (GOBPs) and pheromone-binding proteins (PBPs) are numerically dominant among classic OBPs (Pelosi et al., 2006). PBPs are expressed in long sensillum trichodea and show a male antennae-biased expression pattern. PBPs are involved in the detection of female sex pheromones (Maida et al., 2005; Guo et al., 2012). GOBPs, including GOBP1 and GOBP2, are usually distributed in sensillum basiconica. GOBPs are thought to bind general odorants such as host plant volatiles and other environmental chemical cues (Vogt et al., 1991; Laue et al., 1994). However, a few studies reported that GOBPs may also be involved in sex pheromone detection (Ziegelberger, 1995; Zhou et al., 2009; Liu et al., 2014; Khuhro et al., 2017). For example, competitive fluorescence binding assays showed that GOBPs in *Spodoptera exigua* (Liu et al., 2014) and *Chilo suppressalis* (Khuhro et al., 2017) have high binding affinities for sex pheromones. While GOBPs in *Carposina sasakii* showed high affinities to both host plant volatiles and sex pheromones (Tian et al., 2019). *In situ* hybridization showed that GOBP2 in *Mamestra brassicae* was abundantly distributed in the sensilla responsive to the sex pheromone, *Z*11-16: OH (Jacquin-Joly et al., 2000). In *Plutella xylostella*, both PxylGOBP1 and PxylGOBP2 strongly bind to the sex pheromone *Z*11-16: Ald (Zhu et al., 2016). On the other hand, in *Athetis lepigone*, AlepGOBP2 could bind to the insecticides, chlorpyrifos and phoxim (Zhang et al., 2021). Therefore, the exact function of GOBPs in olfaction is still a conundrum.

The variegated cutworm *Peridroma saucia* Hübner (Lepidoptera: Noctuidae) is a polyphagous feeder on various vegetables and field crops. Generally, *P. saucia* larvae sporadically damage crops during the growing season and pose great problems in the mid-summer (Rings et al., 1976). This pest is endemic to North America and Europe (Struble et al., 1976; Simonet et al., 1981; Willson et al., 1981). Since the 1970s, *P. saucia* has invaded Korea and Japan and gradually become an important pest worldwide (Inomata et al., 2002; Choi et al., 2009). In China, the first outbreak of *P*. *saucia* was reported in Sichuan Province in 1985 (Kuang, 1985). It has spread to more than 13 provinces in China in recent years (Li et al., 2007; Guo et al., 2010; Xuan et al., 2012; Sun et al., 2020). The female sex pheromone components of *P*. *saucia* are *Z*11-16: Ac and *Z*9-14: Ac. The mixture of *Z*11-16: Ac and *Z*9-14: Ac (3:1) could efficiently attract male *P. saucia* moths in the field (Inomata et al., 2002; Choi et al., 2009). Our previous research demonstrated that PsauGOBP1 displays high binding affinities to host plant volatiles but not the sex pheromone components (Sun et al., 2021).

In the current study, we conducted a transcriptome analysis of OBPs in *P*. *saucia* to compare the abundance of candidate genes between male and female antennae. A highly abundant OBP, PsauGOBP2, was cloned and then expressed in *Escherichia coli*. Binding affinities of the recombinant PsauGOBP2 to host plant volatiles and female sex pheromone components were tested. Finally, 3D structural modeling and molecular docking were conducted to predict key amino acid residues for ligand binding. The results not only provide new insights into the function of lepidopteran GOBPs but also are helpful for the development of olfaction-based management approaches for *P. saucia*.

## Materials and methods

### Insect rearing and tissue collection

*P*. *saucia* larvae were collected from Luoyang, Henan Province, China, and were reared in an incubator under 16  h L: 8 h D cycle at 23°C ± 1°C and 60% relative humidity. Larvae were fed an artificial diet, and adults were provided with 10% sugar water (Choi et al., 2009). For transcriptome sequencing, male and female antennae were collected separately from 80 individuals of 2-3-day-old adult *P*. *saucia*. For real-time quantitative PCR (RT-qPCR), male and female antennae, mouthparts, and legs were collected separately from 50 to 80 individuals of 2-3-day-old adult *P*. *saucia*. All of the tissue samples were kept in a −80°C freezer until used.

### Transcriptome sequencing

Total RNA from male and female antennae was extracted using Trizol reagent (Invitrogen, Carlsbad, CA, United States). The purity and quantity of the RNA were evaluated with an ND-2000 spectrophotometer (Nanodrop, Wilmington, DE, United States). The RNA integrity was further checked with a 2100 bioanalyzer (Agilent, Santa Clara, CA, United States). Genomic DNA was eliminated from the total RNA with DNase I (Takara, Beijing, China). mRNA was then isolated from ≥1 µg (concentration ≥50 ng/μL) of the total RNA with Dynabeads mRNA purification kit (Invitrogen, United States). RNA-seq libraries were constructed following Illumina’s library construction protocol and then sequenced on the Nova seq6000 platform (Illumina, United States) at Origingene, Shanghai, China. Sequence assembly was performed with a *de novo* method (Trinity v 2.11) as we previously described (Sun et al., 2020). Putative OBP transcripts were retrieved from the obtained unigenes by searching a pooled non-redundant database using BLASTX algorithm-based method (E-value < 1e-5). Open reading frames (ORFs) of the transcripts encoding candidate PsauOBPs were predicted with ORFfinder (https://www.ncbi.nlm.nih.gov/orffinder). To evaluate the expression abundance, TPM values (Transcripts Per Kilobase of exon model per Million mapped reads) of candidate PsauOBP transcripts were calculated with Salmon (v 1.4.0).

### Phylogenetic analysis of OBPs

A neighbor-joining tree of candidate PsauOBPs and homologs from other lepidopteran species including *Bombyx mori*, *Spodoptera litura*, *Helicoverpa armigera*, and *Agrotis ipsilon* was constructed with MEGA 11. The evolutionary distance was calculated with the JTT matrix-based method (Jones et al., 1992). Node supports of branches were evaluated with a bootstrap method of 1,000 replicates. The constructed tree was visualized and edited in FigTree (v 1.4.2). Amino acid sequences of OBPs used in the phylogenetic analysis were listed in Supplementary Table S1.

### Gene cloning and RT-qPCR of PsauGOBP2

PCR amplification of PsauGOBP2 was carried out with Premix Taq (Takara) under the following procedure: 94°C for 3 min; 34 cycles of 94°C for 20 s, 55°C for 30 s, and 72°C for 1 min. The crude PCR products were then ligated into a pGEM-T easy vector (Promega, Beijing, China) at a molar ratio of 5: 1 (insert: plasmid). The ligation products were used to transform *E*. *coli* Top10 cells. Positive colonies were selected by the T7 and SP6 primers. The colonies were then grown in LB liquid medium and custom sequenced at Origingene, Shanghai, China. The signal peptide of PsauGOBP2 is predicted with SignalP (v 5.0); Protein parameters including the molecular weight and the theoretical isoelectric point were predicted with ProtParam (https://web.expasy.org/cgi-bin/protparam/protparam). Gene-specific primers designed against the PsauGOBP2 cDNA were listed in Supplementary Table S2.

RT-qPCR of PsauGOBP2 in different chemosensory tissues was performed using a Roche LightCycler 480 System (F. Hoffmann-La Roche Ltd., Basel, Switzerland) with a mixture (final volume 20 µL) of 10 μL of TB Green Fast qPCR Mix (Takara), 0.8 μL (10 μM) of each primer, 5 ng of sample cDNA, and appropriate volume of sterilized ultrapure H_2_O. The RT-qPCR program was set as: 1 cycle of 94°C for 30 s; 40 cycles of 94°C for 5 s and 60°C for 10 s; followed by 1 cycle of 95°C for 5 s, 60°C for 1 min, and 50°C for 30 s. The primers designed with Primer Premier 6.0 were listed in Supplementary Table S2. Expression levels of PsauGOBP2 in different tissues were normalized with the endogenous gene *Psauβ*-*actin* (accession number QQ472022), using the 2^−ΔΔCT^ method (Livak and Schmittgen, 2001). Three biological replications were performed for each tissue sample, and each biological replication was performed with three technical replicates. The data obtained from different samples were analyzed with a one-way analysis of variance (ANOVA) followed by the Tukey multiple comparison test.

### Expression and purification of recombinant PsauGOBP2

For the expression of recombinant PsauGOBP2, pET-30b containing the sequence encoding mature PsauGOBP2 was used to transform BL21 *E*. *coli* cells. Protein expression was induced by the addition of IPTG (final concentration 0.4 mM) when the OD_600_ value of cell culture reached about 0.8. Cells cultured for further 2–4 h at 37°C were harvested by centrifugation. PsauGOBP2 was present as inclusion bodies. 10 mL of 8 M urea and 1 mM DTT in Tris buffer (50 mM, pH 7.4) were then used to solubilise the inclusion body pellet (from 1 L of culture). The dissolved protein was dialyzed against 1 L of Tris buffer (50 mM, pH 7.4). The recombinant protein was purified on the anion exchange resins QFF, following standard protocols previously adopted for other moth OBPs (Sun et al., 2012; Sun et al., 2021).

### Fluorescence measurements

The fluorescence binding assays were conducted on a Hitachi F-2710 with a 1 cm light path quartz cuvette. To measure the affinity of the fluorescent probe 1-NPN (N-phenyl-1-naphthylamine) to PsauGOBP2, a 2 mM solution of the protein in 50 mM Tris-HCl, pH 7.4, was titrated with aliquots of 1 mM 1-NPN in methanol to a final concentration of 20 µM. The probe was excited at 337 nm, and emission spectra were recorded between 380 and 460 nm. The dissociation constant of 1-NPN (K_1-NPN_) to PsauGOBP2 was obtained by processing the data with GraphPad Prism 6.0.

Binding affinities of odorants to PsauGOBP2 were measured by competitive binding assays. A panel of 28 compounds (competitors) including the *P*. *saucia* female sex pheromone components *Z*11-16: Ac and *Z*9-14: Ac and 26 host plant volatiles were used in the assay. The CAS number, source, and purity of these compounds were listed in Supplementary Table S3. A solution of PsauGOBP2 and 1-NPN, both at the concentration of 2 mM, was titrated with 1 mM of each competitor (dissolved in methanol) at a final concentration of 10 µM (sex pheromones) or 20 µM (host plant volatiles). The dissociation constants (K_i_) of competitors to PsauGOBP2 were calculated using the equation: K_i_ = [IC_50_]/(1 + [1-NPN]/K_1-NPN_), where [IC_50_] is the concentration of the competitor halving (50%) the initial fluorescence value (100%), [1-NPN] is the free concentration of 1-NPN, and K_1-NPN_ is the dissociation constant of the protein-1-NPN complex.

### Structural modelling and molecular docking

A 3D structure of PsauGOBP2 was modeled with Alphafold2 (Jumper et al., 2021). The 3D structure was then evaluated by SAVES (v 6.0). Molecular docking evaluations for PsauGOBP2 with the ligands were performed with AutoDock Vina (v.1.1.2). The default parameters were set as described in the Autodock Vina manual. The top-ranked conformation which was estimated by the Vina docking score was then subjected to PyMOL (v 1.9.0) for visual analyses.

## Results

### Antennal transcriptome sequencing and assembly

Transcriptomic data of *P*. *saucia* antennae was obtained using a Nova seq6000 platform. A total of 44.85 million and 35.53 million clean reads were produced in male and female samples, respectively. All clean reads from male and female data were then merged for *de novo* assembly, which generated a total of 151,541 unigenes with an N_50_ length of 1158 bp and a mean length of 683.39 bp (Supplementary Table S4). Moreover, 27.24% of the unigenes were longer than 1000 bp based on the size distribution analysis.

### Identification of candidate PsauOBPs and phylogenetic analysis

**FIGURE 1:**
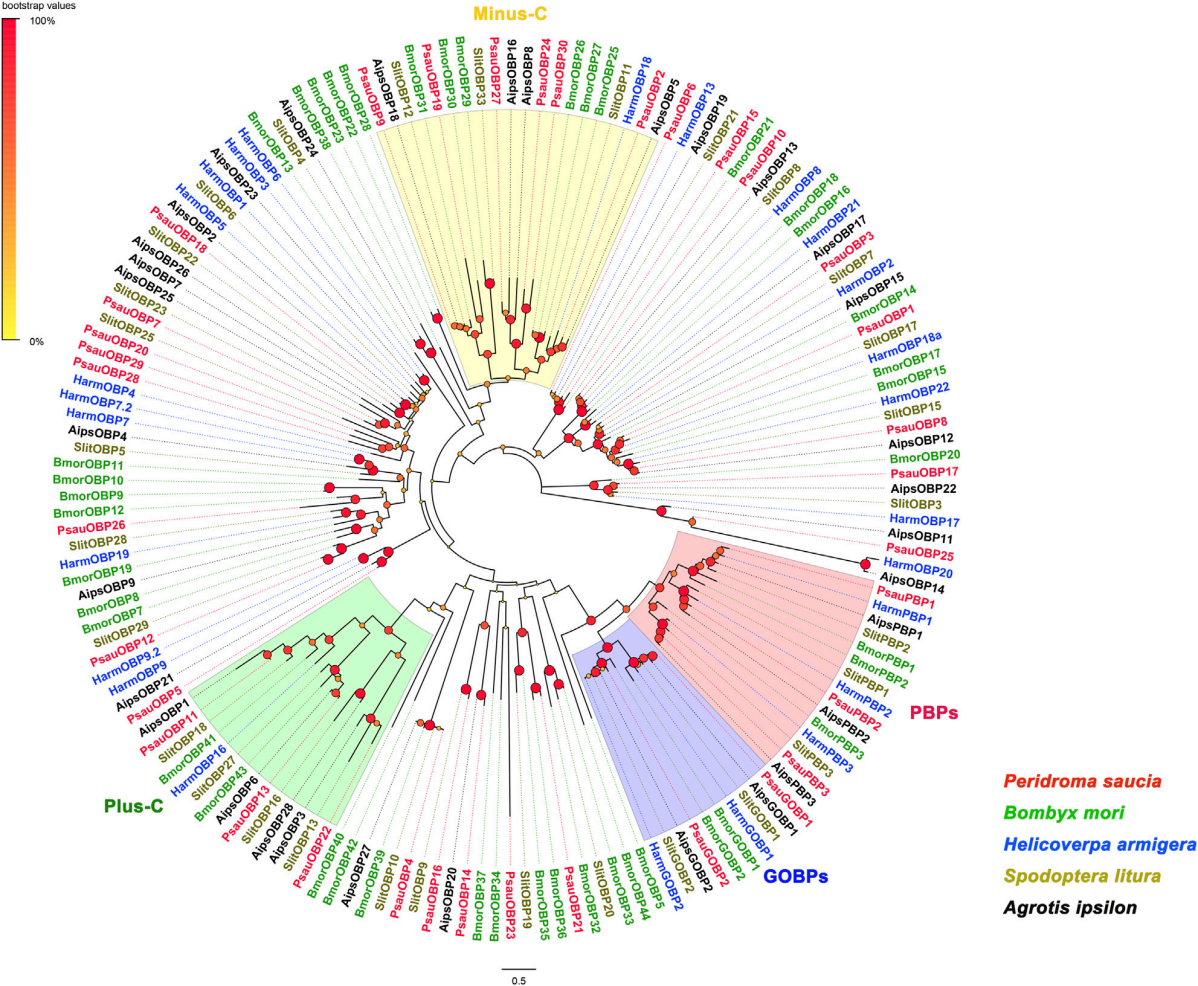
Neighbor-Joining tree of OBPs from *P*. *saucia* and other Lepidoptera species. Node support was estimated with 1000 replicates. The bootstrap values are indicated by the size and color of circles at the branch nodes based on the scale at the top left. The accession numbers of all OBPs used in the phylogenetic analysis are provided in Supplementary Table S1.

By homologous searching with BLASTX, a total of 35 putative OBP transcripts were identified in the transcriptome of *P*. *saucia* antennae (Supplementary Table S5). Except for PsauOBP19, PsauOBP24, PsauOBP26, and PsauOBP30, the other 31 PsauOBPs have full length ORFs based on the presence of start codons, stop codons, and the BLASTP alignment results to other homologous OBPs. Phylogenetic analysis of 156 OBPs from different lepidopteran species including *P*. *saucia* (this study), *B. mori*, *H*. *armigera*, *S*. *litura*, and *A*. *ipsilon* revealed several distinct clades, where three putative PsauPBPs (PsauPBP1/2/3) were clustered with PBPs from other species; PsauGOBP1 and PsauGOBP2 were grouped with other GOBP1s and GOBP2s, respectively (Figure 1). Furthermore, we found that GOBPs share one single original lineage with PBPs (Figure 1), consistent the reports for other lepidopteran species (Vogt et al., 2015). Of the 35 PsauOBPs, 23 were classic OBPs characteristic of six conserved cysteines. Six OBPs (PsauOBP2/9/19/24/27/30) with 4 conserved cysteines were clustered into the minus-C OBP clade. PsauOBP11, PsauOBP13, and PsauOBP22 possessed two extra conserved cysteines and accordingly were clustered into the plus-C OBP clade (Figure 1; Supplementary Table S5). By contrast, PsauOBP14, PsauOBP16, and PsauOBP25 did not match any classification based on the significantly longer sequences (with 258, 337, and 244 amino acid residues, respectively) and the presence of 5 (for PsauOBP25) or 7 (for PsauOBP14 and PsauOBP16) cysteines (Supplementary Table S5).

### TPM value analyses of candidate PsauOBPs

**FIGURE 2:**
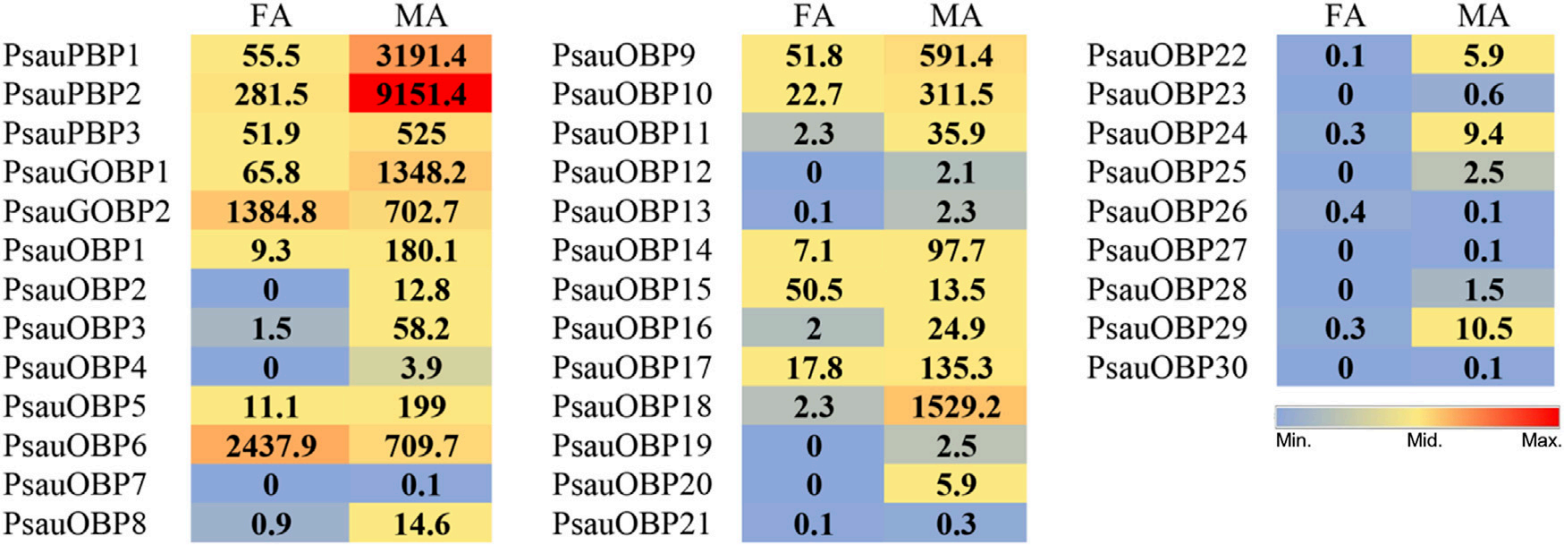
TPM values of candidate *PsauOBPs* in female (FA) and male (MA) antennae.

Transcript abundance analysis based on TPM values showed that seven OBPs, including *PsauPBP1*, *PsauPBP2*, *PsauPBP3*, *PsauGOBP1*, *PsauGOBP2*, *PsauOBP6*, and *PsauOBP18*, had high transcript levels in the antennae with an average TPM value of >500 in male and female samples (Figure 2). Of these, *PsauPBP1* (3191.4/55.5 TPM values for male/female, same below), *PsauPBP2* (9151.4/281.5), *PsauPBP3* (525/51.9), *PsauGOBP1* (1348.2/65.8), and *PsauOBP18* (1529.2/2.3) showed higher levels in males than in females. While *PsauOBP6* and *PsauGOBP2* showed higher TPM values in female (2437.9 and 1384.8, respectively) than in male antennae (709.7 and 702.7, respectively). Other *PsauOBPs* showed relatively low TPM values and their expressions were variable in male and female antennae (Figure 2).

### Gene cloning and sequence analysis of PsauGOBP2

**FIGURE 3:**
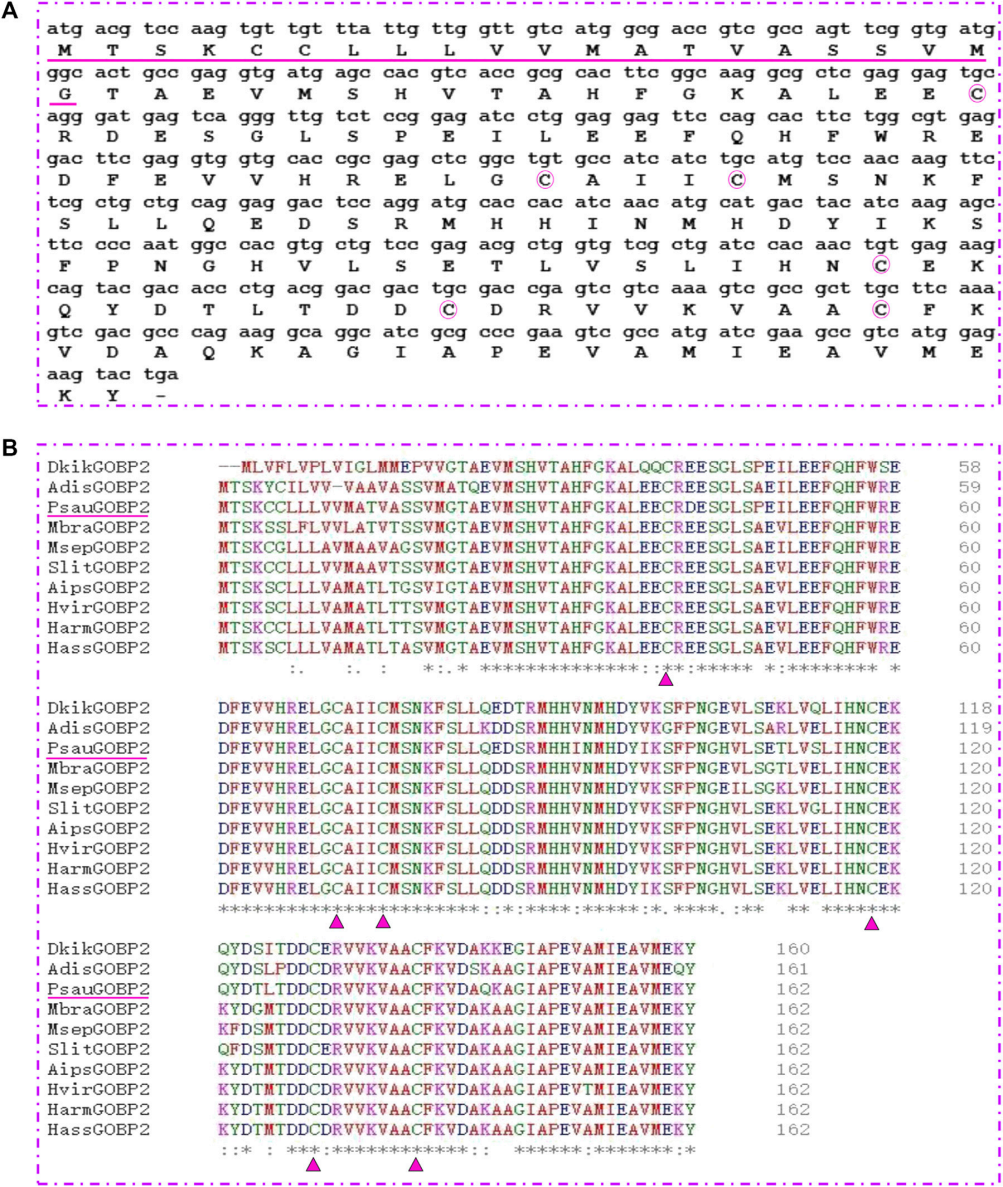
Sequence characterization of PsauGOBP2. **(A)** cDNA sequence and deduced amino acid sequence of the PsauGOBP2. The predicted signal peptide is underlined. The six conserved cysteines are circled in pink. **(B)** Alignment of PsauGOBP2 with orthologs from other lepidopteran species. *Dendrolimus kikuchii* (DkikGOBP2, AGJ83353.1); *Athetis dissimilis* (AdisGOBP2, ALJ93807.1); *Mamestra brassicae* (MbraGOBP2, AAC05703.2); *Mythimna separata* (MsepGOBP2, AWT22242.1); *Spodoptera litura* (SlitGOBP2, XP_022817877.1); *Agrotis ipsilon* (AipsGOBP2, AAP57462.1); *Heliothis viresence* (HvirGOBP2, PCG76987.1); *Helicoverpa armigera* (HarmGOBP2, CAC08211.1); *Helicoverpa assulta* (HassGOBP2, AAQ54909.1). The six conserved cysteine residues in the GOBP2s are indicated with pink triangle. Residues with similar physicochemical properties are shown with “.” and “**:**”; Identical residues are indicated with “*”.

Based on the TPM values, we focused on a highly abundant transcript in the antennae of *P*. *saucia*, PsauGOBP2. First, the full length of *PsauGOBP2* was amplified from the *P*. *saucia* antennae. The ORF of *PsauGOBP2* is 489 bp encoding 162 amino acids, and the predicted matured PsauGOBP2 contains 141 amino acids (Figure 3A). The molecular weight of the mature protein is 16.1 kDa with an isoelectric point of 5.06. The amino acid sequence of PsauGOBP2 has the six-cysteine signature that forms the motif C_1_-X_25-30_-C_2_-X_3_- C_3_-X_36-42_-C_4_-X_8-14_-C_5_-X_8_-C_6_, a typical feature of classic OBPs. Further multiple alignments revealed distinct sequence similarities between PsauGOBP2 and other lepidopteran GOBP2s (Figure 3B). PsauGOBP2 exhibited the highest identity with SlitGOBP2 of *S. litura* (90.12%), followed by AipsGOBP2 of *A. ipsilon* (88.89%).

### Expression profiling of PsauGOBP2

**FIGURE 4:**
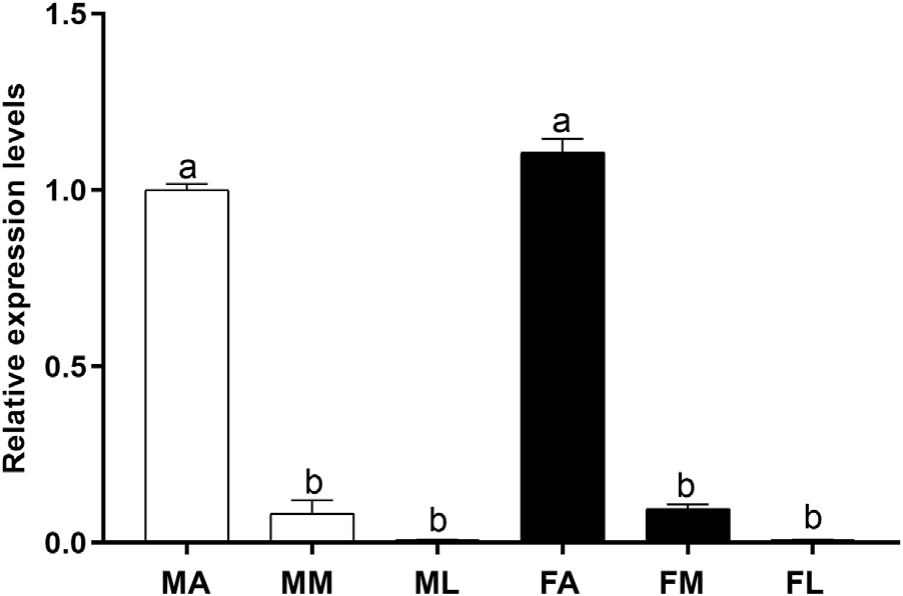
RT-qPCR of *PsauGOBP2* in different chemosensory tissues of *P*. *saucia*. MA: male antennae; MM: male mouthparts; ML: male legs; FA: female antennae; FM: female mouthparts; FL: female legs. Means (+SE) with different letters are significantly different (*p* < 0.05) according to a one-way ANOVA followed by Tukey multiple comparison test, n = 3.

To investigate the expression profile of *PsauGOBP2* in *P*. *saucia*, we measured its transcript levels in different chemosensory tissues including antennae, mouthparts, and legs of both sexes. RT-qPCR results showed that the expression of *PsauGOBP2* was significantly higher in the antennae than in other tissues. Moreover, *PsauGOBP2* expression was slightly higher in female antennae than in male antennae; however, the difference was not significant (Figure 4).

### Prokaryotic expression and purification of PsauGOBP2

**FIGURE 5:**
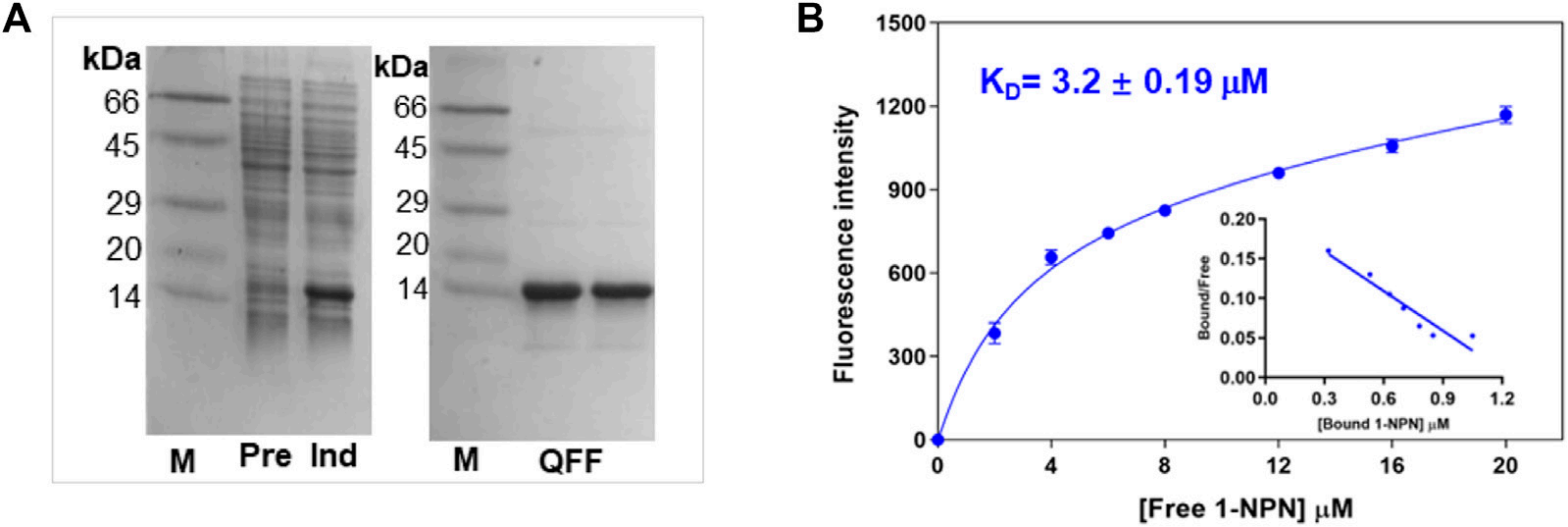
Preparation of the recombinant PsauGOBP2 and its affinity with the fluorescence probe 1-NPN. **(A)** SDS-PAGE analysis of the crude bacterial extracts before (Pre) and after (Ind) induction with IPTG, and purification of the recombinant PsauGOBP2 on the QFF column. **(B)** Affinity of PsauGOBP2 to 1-NPN. Analysis of the fluorescence values (means +SE, n = 3) with GraphPad Prism 8 software indicated the presence of a single binding site with the K_1-NPN_ value of 3.2 µM.

To obtain the recombinant protein, *PsauGOBP2* encoding mature protein was cloned and ligated into the expression vector pET-30b. As shown in Figure 5A, the recombinant PsauGOBP2 was abundantly expressed in the transformed *E*. *coli* BL21 cells when induced with IPTG. After purification with anion exchange resins, an expected size of the target protein was obtained (Figure 5A). The purified protein was then used in the fluorescence binding assays.

### Ligand binding affinities of PsauGOBP2

**FIGURE 6:**
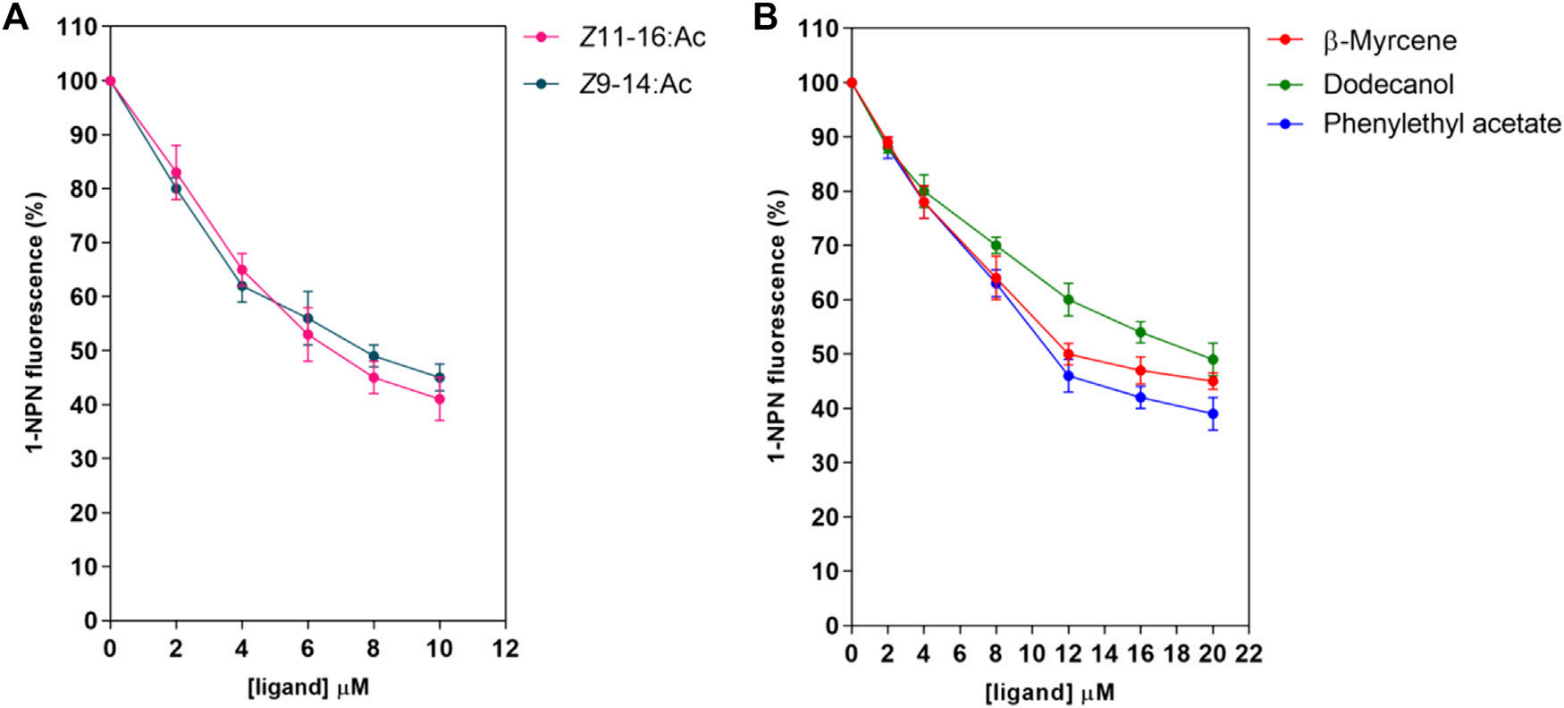
Competitive fluorescence binding assays of selected ligands to the recombinant PsauGOBP2. **(A)** *P*. *saucia* female sex pheromone components; **(B)** selected host plant volatiles. Affinities of the sex pheromone components of female *P*. *saucia* and 26 host plant volatiles were analyzed. Detailed information for all of the tested compounds is reported in Supplementary Table S3, S6.

First, the binding pocket of recombinant PsauGOBP2 was saturated by the fluorescent probe 1-NPN, resulting in a K_1-NPN_ value of 3.2 ± 0.19 μM (Figure 5B). Then 28 ligands including *P*. *saucia* female sex pheromone components and host plant volatiles were used as competitors to displace the probe from the binding pocket. The results indicated that the sex pheromone components *Z*11-16: Ac and *Z*9-14: Ac were the strongest ligands, with the K_i_ values of 4.2 ± 0.8 μM and 4.9 ± 0.6 μM, respectively (Figure 6A; Supplementary Table S6). Three host plant volatiles, phenylethyl acetate, β-myrcene, and dodecanol also showed binding affinities to PsauGOBP2, with the K_i_ values of 6.3 ± 0.3 μM, 8.0 ± 0.3 μM, and 13.0 ± 0.4 μM, respectively (Figure 6B; Supplementary Table S6). The other tested compounds showed no affinities to PsauGOBP2 because the IC_50_ values were higher than 30 μM (Supplementary Table S6).

### Protein structure modeling and molecular docking

**FIGURE 7:**
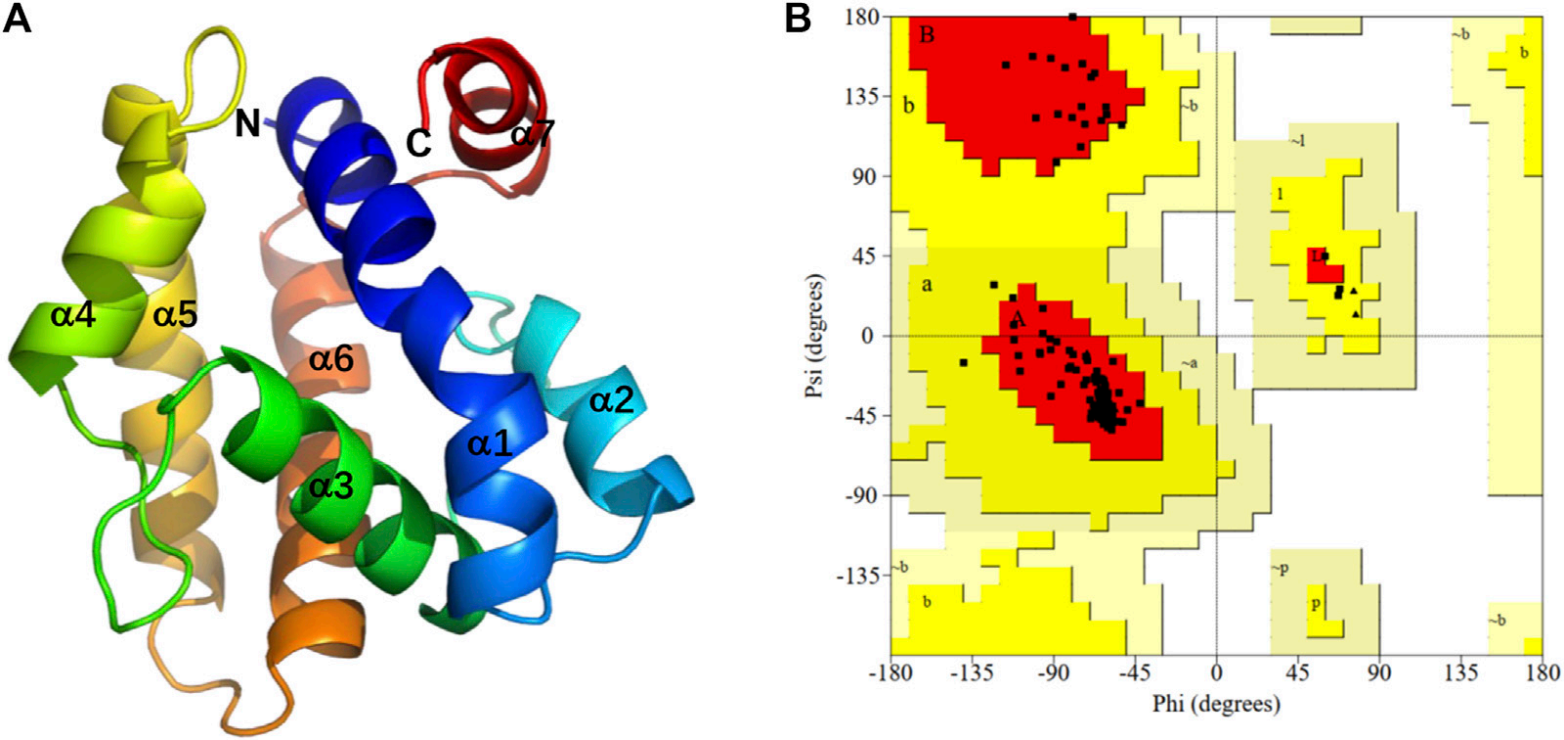
Three-dimensional structural analysis of PsauGOBP2. **(A)** Predicted 3D structure of the PsauGOBP2; **(B)** Ramachandran plot showing residue compatibilities and stereochemical rationalities of the model. A, B, L: residues in most favored regions. a, b, l, p: residues in additional allowed regions. ∼a, ∼b, ∼l, ∼p: residues in generously allowed regions.

As the crystal structure of PsauGOBP2 has not yet been resolved, we applied a highly accurate modeling program, Alphafold2, to build a 3D structure of PsauGOBP2 (Figure 7A). The model evaluation demonstrated that sequence identities between the residues with queries were >80%, the pLDDT score (per-residue predicted local-distance difference test) was >90%, and the PAE value (predicted aligned error) was approximately equal to 0 Å (Supplementary Figure S1). Further PROCHECK evaluation demonstrated that 100% of the non-glycine and non-proline residues were trapped in the allowing areas and 95.4% of the amino acid residues were located in the most favored areas (Figure 7B). These results indicated that the predicted model of PsauGOBP2 was reliable and qualified for further analysis.

**FIGURE 8:**
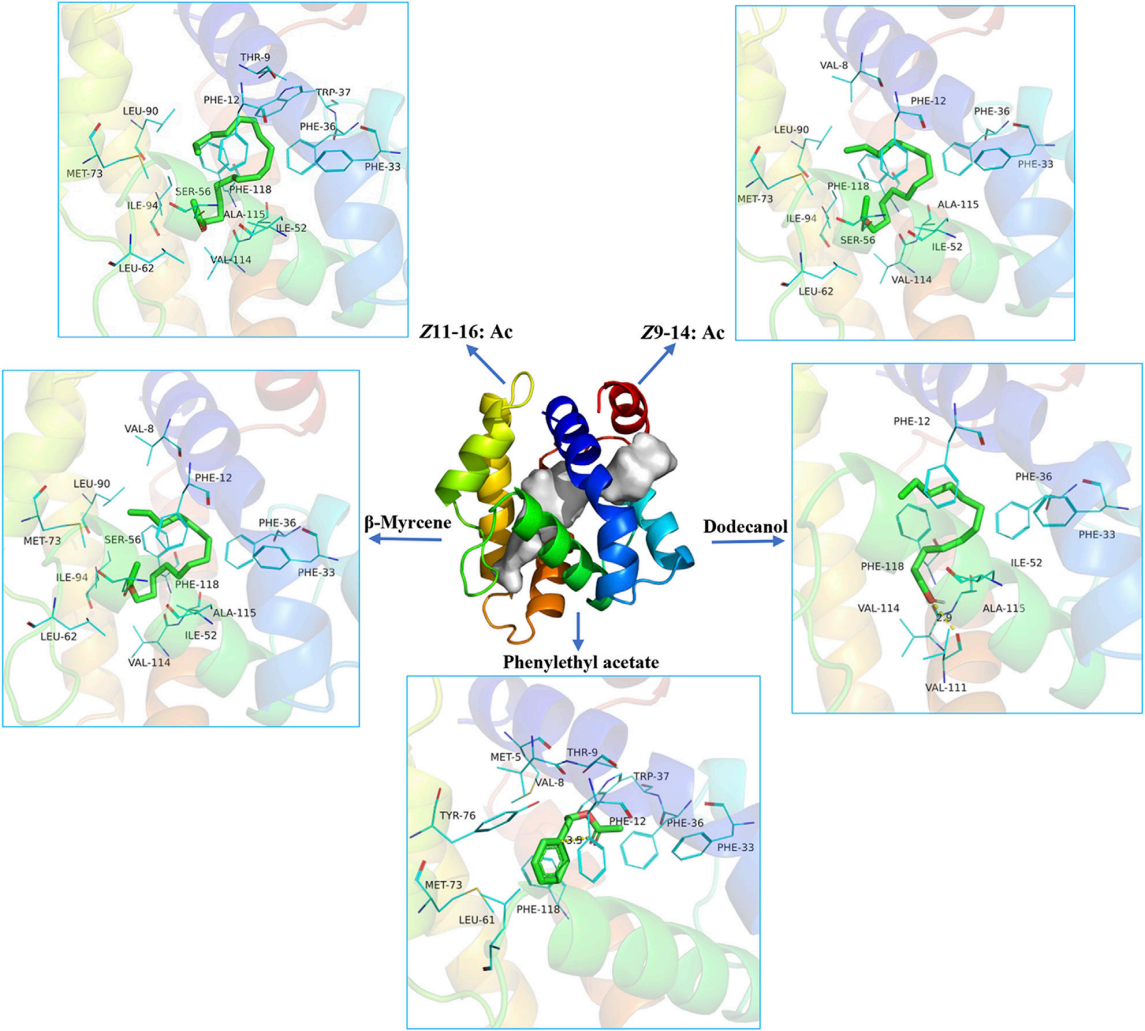
Molecular docking of the ligands in the binding site of PsauGOBP2.

The structure prediction with Alphafold2 revealed that PsauGOBP2 comprised seven α-helixes (α1–α7) (Figure 7A), which is reminiscent of the structure of other moth GOBPs (Zhou et al., 2009; Zhou, 2010). Binding energy analysis showed that the docking binding energy between PsauGOBP2 and each ligand was ≤ −6 kcal.mol^−1^ and the distances of all potential interactive residues were <4 Å. Furthermore, the molecular docking analysis found several residues in PsauGOBP2 involved in the binding with more than one ligand. Four aromatic amino acid residues, i.e., Phe-12, Phe-33, Phe-36, and Phe-118, were needed for the binding to *Z*11-16: Ac, *Z*9-14: Ac, phenylethyl acetate, β-myrcene, and dodecanol; three nonpolar amino acid residues, Ile-52, Val-114, and Ala-115, for *Z*11-16: Ac, *Z*9-14: Ac, β-myrcene, and dodecanol; two polar amino acid residues, Thr-9 for *Z*11-16: Ac and phenylethyl acetate and Ser-56 for *Z*11-16: Ac, *Z*9-14: Ac, and phenylethyl acetate (Figure 8, Table 1). Notably, the hydrogen bond (2.9 Å) and conjugated bond (3.5 Å) mediated the binding of PsauGOBP2 to dodecanol and phenylethyl acetate, respectively (Figure 8).

**TABLE 1:** Putative key amino acid residues in the docking of PsauGOBP2 to the ligands.

Ligand	Key amino acid residues	
	Nonpolar	Polar
*Z*11-16: Ac	Phe-12, Phe-33, Phe-36, Trp-37, Ile-52, Leu-62, Met-73, Leu-90, Ile-94, Val-114, Ala-115, Phe-118	Thr-9, Ser-56
*Z*9-14: Ac	Val-8, Phe-12, Phe-33, Phe-36, Ile-52, Leu-62, Met-73, Leu-90, Ile-94, Val-114, Ala-115, Phe-118	Ser-56
β-Myrcene	Val-8, Phe-12, Phe-33, Phe-36, Ile-52, Leu-62, Met-73, Leu-90, Ile-94, Val-114, Ala-115, Phe-118	Ser-56
Phenylethyl acetate	Met-5, Val-8, Phe-12, Phe-33, Phe-36, Trp-37, Leu-61, Met-73, Phe-118	Thr-9, Tyr-76
Dodecanol	Phe-12, Phe-33, Phe-36, Ile-52, Val-111, Val-114, Ala-115, Phe-118	—

“—”means no candidate polar residues were found for the binding of the ligand with PsauGOBP2.

## Discussion

Understanding of how insects sense external chemical stimulants is important for developing effective pest management strategies. OBPs represent the first step of odorant recognition in insect chemical communication (Laughlin et al., 2008; Brito et al., 2016; Rihani et al., 2021; Han et al., 2022). In the current study, we used transcriptome sequencing to identify OBPs in the antennae of *P*. *saucia*. Then we investigated the expression profile, ligand affinity, and binding mechanism of PsauGOBP2.

In this study, we identified a repertoire of 35 OBPs in the antennal transcriptome of *P*. *saucia*. This number is close to that identified in the antennae of other noctuid moths such as *H*. *armigera*, *Mythimna separata*, *S*. *litura* and *S*. *exigua*, which have 34, 32, 38, and 45 OBPs, respectively (Gu et al., 2015; Zhang et al., 2015; Chang et al., 2017; Du et al., 2018). Of the OBPs identified in *P*. *saucia*, 3 are plus-C OBPs and 6 are minus-C OBPs, which is in accordance with the reported 3 to 6 plus-C/minus-C OBPs in other moths (Gu et al., 2015; Zhang et al., 2015; Chang et al., 2017; Du et al., 2018).

According to TPM values, PsauGOBP2 was abundantly expressed in both male and female antennae. Hence, we further explored the expression profiles and binding abilities of PsauGOBP2. According to the RT-qPCR result, the expression levels of *PsauGOBP2* between male and female moths are similar, albeit slightly higher in female antennae. The inconsistency between TPM values and expression levels could be explained by that TPM values are rough estimates of gene transcript levels based on the calculation of transcripts per million mapped reads. Therefore, gene expression levels in different tissues need to be validated by RT-qPCR. Similar findings were reported in *S*. *litura* where *SlitGOBP2* showed similar expression levels between male and female antennae (Liu et al., 2015). However, GOBP2 in *A*. *ipsilon* is female antennae-biased (Huang et al., 2018), and GOBP2s from *Maruca vitrata* and *Chilo suppressalis* show significantly higher expression levels in male antennae than in female antennae (Zhou et al., 2015; Khuhro et al., 2017). Such differences might be an adaption to species-specific chemical environments, reflecting olfaction plasticity in insects (Gadenne et al., 2016).

Lepidoptera PBPs and GOBPs form a monophyletic lineage with a single ancestral origin. They have undergone divergence by gene duplication under different selection pressures (Vogt et al., 2015). Numerous studies indicated that PBPs selectively bind sex pheromones and contribute to long-distance mate recognition in moths (Guo et al., 2012; Han et al., 2022; Zhong et al., 2022). In contrast, GOBP1s and GOBP2s are thought to bind host plant volatiles and sex pheromones, respectively (Jacquin-Joly et al., 2000; Gong et al., 2009; Khuhro et al., 2017; Huang et al., 2018; Zhang et al., 2020). To determine the binding abilities of PsauGOBP2, we selected 28 compounds for fluorescence binding assays. These compounds include the volatiles emitted by soybean, maize, cotton, and tobacco (Knudsen et al., 1993; Loughrin et al., 1994; Boué et al., 2003; Yan et al., 2005; Yan and Wang, 2006), and sex pheromone components (*Z*11-16: Ac and *Z*9-14: Ac) of female *P*. *saucia* (Inomata et al., 2002; Choi et al., 2009). Our results demonstrated that PsauGOBP2 has high binding affinities (K_i_ < 5 µM) with *Z*11-16: Ac and *Z*9-14: Ac and moderate binding affinities (6 µM ≤ K_i_ ≤ 13 µM) with the host plant volatiles phenylethyl acetate, β-myrcene, and dodecanol. Meanwhile, our previous research validated that PsauGOBP1 can actively bind the host plant volatiles (*Z*)-3-hexenyl acetate (K_i_ = 4.0 µM), citral (K_i_ = 5.6 µM), farnesol (K_i_ = 6.4 µM), nonanal (K_i_ = 6.8 µM) (*Z*)-3-hexen-1-ol (K_i_ = 8.5 µM), and benzaldehyde (K_i_ = 9.4 µM) (Sun et al., 2021). Therefore, we suggested that PsauGOBP2 plays important roles in the detection of sex pheromones in *P*. *saucia*, while PsauGOBP1 mainly participates in the recognition of host plants. This inference needs to be validated with *in vivo* analyses, such as gene knockdown/out combined with behavioral investigation. Moreover, comparative studies of the function of PsauGOBP2 and PsauPBPs in sex pheromone detection will be an important aspect of our future studies. Notably, our results are in agreement with binding abilities of BmorGOBP1 and BmorGOBP2 in *B*. *mori*, though BmorGOBP1 also shows relatively low affinities with the sex pheromones (Zhou et al., 2009). Similar results were reported for GOBP1s and GOBP2s in *S*. *litura*, *A*. *ipsilon*, and *A*. *lepigone* (Liu et al., 2015; Huang et al., 2018; Zhang et al., 2020). Female sex pheromone components of *S*. *litura* are *Z*9-14: Ac, *E*11-14: Ac, *Z*9, *E*11–14: Ac, and *Z*9, *E*12-14: Ac (Tamaki et al., 1973; Sun et al., 2002; Wei et al., 2004). Of which, *Z*9-14: Ac is also one of the female sex pheromone components of *P*. *saucia*. Like PsauGOBP2, SlitGOBP2 could strongly bind *Z*9-14: Ac with high affinities (Liu et al., 2015), suggesting that GOBP2 is functionally conserved in these two insect species. By contrast, in *M*. *vitrata*, GOBP2 only binds to the host plant volatiles (Zhou et al., 2015). Further *in vivo* investigation, such as the application of RNAi or CRISPR/Cas9 system, is needed for the determination of the functions of GOBP2s. Furthermore, as indicated by the RT-qPCR and the TPM values, PsauGOBP2 is highly expressed in both male and female antennae, implying that female moths may also have the ability to detect the sex pheromones released by itself or other female moths. In the future, electroantennogram (EAG) and behavioral responses of the female moths to the sex pheromone components are needed, which are useful to fully understand the functional roles of GOBP2s in moths (Bjostad, 1998).

Previous studies have demonstrated that insect OBPs bind specific ligands with polar and nonpolar residues in a hydrophobic cavity (Tegoni et al., 2004; Sun et al., 2013; Liu et al., 2019; Li et al., 2021). In molecular docking analysis, the docked binding energy between PsauGOBP2 and *Z*11-16: Ac, *Z*9-14: Ac, phenylethyl acetate, β-myrcene, and dodecanol was −6.9 kcal.mol^-1^, −6.7 kcal.mol^-1^, −6.5 kcal.mol^-1^, −6.1 kcal.mol^-1^, and −6.0 kcal.mol^-1^, respectively. This is in accordance with the results of the fluorescence competitive binding assay which showed that the K_i_ value for each ligand was 4.2 μM, 4.9 μM, 6.3 μM, 8.0 μM, and 13.0 μM, respectively. Moreover, we found several key polar and nonpolar amino acid residues involved in the binding of PsauGOBP2 to the ligands, as reported for other insect OBPs (Tegoni et al., 2004; Sun et al., 2013; Liu et al., 2019; Li et al., 2021). Furthermore, some residues, such as Thr-9, Phe-12, Phe-33, Phe-36, Ile-52, Ser-56, Val-114, Ala-115, and Phe-118, can interact with more than one ligand, indicating that these residues might play prominent roles in the ligand recognition of PsauGOBP2. Further investigation involving site-directed mutagenesis assays is needed to validate the necessity of these residues in the binding of PsauGOBP2 to the five ligands. Of which, site-directed mutagenesis of two predicted polar residues, Thr-9 and Ser-56, is especially needed to understand the binding mechanism of PsauGOBP2 to *Z*11-16: Ac. Of note, we did not find putative polar residues for the binding to dodecanol. This may be due to the characteristics of the compound and/or the parameter we set for the docking analysis. If the we set potential interaction distance to be <6 Å (but not <4 Å), we could spot some polar amino acid residues that are possibly involved in the binding of PsauGOBP2 to dodecanol.

In summary, our studies provide the expression pattern of OBPs in the antennae of *P*. *saucia*. Among the OBPs, PsauGOBP2 is abundantly expressed in the antennae of both sexes. *In vitro* fluorescence binding assays demonstrated that PsauGOBP2 binds to sex pheromone components as well as some host plant volatiles. Finally, 3D structural modeling and molecular docking showed several amino acid residues in PsauGOBP2 that are involved in ligand binding. The results increase our understanding of the olfactory system of *P*. *saucia* and provide insights into the function and binding mechanism of PsauGOBP2 which would be used as a target for developing olfaction-based management of *P*. *saucia*.

## Data Availability

The datasets presented in this study can be found in online repositories. The names of the repository/repositories and accession number(s) can be found in the article/Supplementary Material.
