# Supplementary material for: Antennal transcriptome analysis of odorant-binding proteins and characterization of GOBP2 in the variegated cutworm Peridroma saucia
Source: Front Physiol. 2023 Aug 10;14:1241324. doi: 10.3389/fphys.2023.1241324 (PMC10450149; doi:10.3389/fphys.2023.1241324)
Supplement: Supplementary file 1 [file DataSheet1.zip › Data Sheet 1/Supplementary materials/Table S1 (sequences for tree).docx]

Table S1. Amino acid sequences of OBPs for phylogenetic analysis.

>PsauPBP1

MAPHPSATMSVRLALVVLAGLFITVECSQEVMKNLSIHFAKPLEDCKKEMDLPDSVATDFVNFWKEGYEFTSRQTGCAIMCVSSKLELLDPEYKLHHGKAQEFAQKHGADEAMAKQLVEMLHGCAQSTPDVADDPCMKTLNVAKCFKAKIHELNWAPDVELLMGEVLAEV

>PsauPBP2

MADSRWRLACFVCVLFATSSVMASKELISKMSQGFAKVVDQCKTELNIGEHIMQDMYNFWREEYALVNRDLGCMVLCMANKLDLIGEDQKMHHGKAEEFAKSHGADEAVAKQLVGILHGCETQHQGIEDPCAMALEVAKCFRTKMHELNWAPSMEVAIEEIMTAV

>PsauPBP3

MGTNHVFLALVLMAVGVREIEPSKDAMKYITSGFVKVLEQCKQELNMTDQILADLFHYWKLDYTLLNRDTGCAIICMSKKLDLLDANGRMHHGNAQEFALKHGAGEQVASQIVTIIHECEKKFERDDDECLRVLEVAKCFRSGIHELDWQPKVEVIVTEVLTEM

>PsauGOBP1

MMQAVLVLVLLAAATLADVNVMKDVTLGFGQALDKCRQESDLTEEKMEEFFHFWRDDFKFEHRELGCAIQCMSRHFNLLTDSSRMHHDNTEEFIQSFPNGEVLARQMVSLIHACEKQFDHEEDHCWRILHVAECFKHSCVQHGVAPSMEMMMTEFIMEAEAR

>PsauGOBP2

MTSKCCLLLVVMATVASSVMGTAEVMSHVTAHFGKALEECRDESGLSPEILEEFQHFWREDFEVVHRELGCAIICMSNKFSLLQEDSRMHHINMHDYIKSFPNGHVLSETLVSLIHNCEKQYDTLTDDCDRVVKVAACFKVDAQKAGIAPEVAMIEAVMEKY

>PsauOBP1

MNTYNFQSIFCILCIICLFFGGSYAMTRQQLKNSGKLMKKSCMPKNDVTEEEVGDIEKGKFIESRNVMCYVACIYTMTQLVKNNKLSYEAVIKQVDIMFPTEMRDAVKAAATSCKEIAKKYKDLCEASYWTAKCMYDYDANNFIFP

>PsauOBP2

MKSFFVICVVFVAGVCAANVTLPPTQQDKAQKLASECIKETGVSSEVLAEAKKGHITEDEGLKKFAYCFFKKAGIVDNDGKLNIEESIAKLPPGVNKDDARKVLEGCKSKSGKTPEDTTFEIFKCYHAGAKTHILLSGI

>PsauOBP3

MMNQKRLCLLFIAMFLATGCDAMTRQQMKNSGKILKKNCMNKNQVTEDQIGSIDKGKFVEDKKVMCYIACIFEMTNVIKNNKLNYEASIRQIDLMYPADLKEGAKAAVEKCKDVQKKYKDICEASFYAAKCMYEFNPADFIFA

>PsauOBP4

MVRQIGLLLCCLCVFGISLSDSAISADSESRCRNPPTAPQKIERVITLCQDEIKLSILREALDVIKEEHTMPAQRRRDKREVPFTHDEKRIAGCLLQCVYRKVKAVDGYGFPTLEGLVGLYSDGVNERGYFMAVLEASRECLMKNHDKFSRTVPMDNGRNCDISFDIFECISDRIGEYCGTSGL

>PsauOBP5

MLKLSVVCFSFAVVAVNFRSAHCMSDEDKQAFVAAMKPMAEECGSDCGLTEEDFKKHNKGEDMDPCFKKCIMQKMGLLDDSGKYNREVLHESISEYTGDKDEAKKIQDQLDGCFDANGDNDGDDEESQMKRVDVLFNCLKELKE

>PsauOBP6

MFTGTLPLVLCVLAVAYGGKEKPVFSEEIKEIIQTVHDECVAKTGVAEEDITNCENGIFKEDPKLKCYMFCLMEEASLVDDDGTVDYDMLVSLIPDEYYERTTKMIFSCKHLDTPDKDKCQRAFEVHRCSYGKDPDLYFLF

>PsauOBP7

MFLIFQVAKAQSLRDSLRPVIVACSKEHGVTDADIQAAKDAGSAASIPPCFIACVFKKAGFLNDKGDYDVETGLKNLRQFVKDNEQYNKLSEVAKACSSVKDKVVRDGAAGCERGGLLAGCFLEHQTSVSCHPINFDTSLCQRFPLRSNRKSSLET

>PsauOBP8

MYSGTIIIFSVILMIVSNSTFVNSQMTREQVKNSGKLVKKTCSAKNDLTEDEVKDVDKGKFIEEKNFMCYIACVYKMGQSIKGNTLNHDMMIRQVDMLFPADMKAPVKAAIEHCRGVAKKHKDICEASYWTAKCVYEFDPPNFMFP
>PsauOBP9

MKTLFVFAACILLAQALTDEQKEKLKKHRTECLTETKVEEQLVNKLKGGDYKTESEPLKKYALCMLTKSELMTKDGKFKKDVALAKVPNAADKPTVEKLIEACLANKGNTPHQTAWNYVKCYHEKDPKHAIFL

>PsauOBP10

MLLIEIVKFLILVATCEAMTMKQIKNTGKMIRKSCQPKNNVEDAKIDPIAEGIFIDEKEVKCYMACVMKMANTIKNGKLNFDAAIKQADLLLPDEVKEPAKEAILACKKAADGHKDICDVSFHVTKCIYNHNPGIFYFP

>PsauOBP11

MFKFCVVLAFCVAVCSAAPGGGGTYCGETPSVIYNCLSAPTVVSPDVSSKCQGSKYTNECERLTCVFREAKWLNGDAVDKTKLTAYFDQFEKDHAAWGPAIQHVKTACLGAELKAQGVFLNCPAYDVMHCVLGSFIKHATPAQWSTSATCAYPRAYAAACPVCPDDCFSPQVPIGSCNACYLPPRTA

>PsauOBP12

MCNLFIVFLAICSCVYALTEQELKMEFTKLIMKCNKDSEVDMVELVQLQSYVIPTKTATKCVLACAYKAAEVMNAQGQYDLDHAYKVAEMMKNGDEKRLVNAKKMADICVKVNEVKVSDGEKGCDRAALIFKCTVENAPKFGFKL

>PsauOBP13

MFKYSVFLLFVTASYADLLSQRENKGATLKPLSVCCDIPEMGDPKHLAKCSNPKLPGPCNDVECVFLESGFLTDKNTLNKEAYKNHLKQWEESNKGWTVAVDKAIKECVDNDPRQHLDYPCKAYDVFTCTGIAMLKKCPEAAWKC

>PsauOBP14

MHVCDANFHFRTMVGAVVLFTLLPVWVACSGEGNIKLLEDEVAVALKACTYPDDTVLPKEPVSKERQRRSDDESYDGSPRIDSNMKGGQRYSHERRNNNDSGDQMSIINATDYDYEGYGTGSMGEKLLTSIPRPAAVNNNINNNNTSRTRRSEPLLNKPDTDQCLSQCVFANLQVVDTRGIPREAEVWNKVQASVTSQQSRSALHDQIRACFQELQSEAEDNGCSYSNKLERCLMLRFSDRKVDGKASTQKPVSTEQS

>PsauOBP15

MGFCFRTKSRSVSRCVQYTIILTTFLFQVFSDEIKEIIQTVHDECVSKTGVAEEDITNCENGIFKEDVKLKCYMFCLMEEASLVDENDVVDYDLLVSLIPDEYYDRTSNMIFACKHLDTPDKDKCQRAFDVHKCSYDKDPALYFLF

>PsauOBP16

MCLVKYHVLILCLIIVESYALNCRSSGGPKEAELKNIYKKCLKMQEGKNSSKGNSEQDYKEPRGQNIQRSDWERGRSTGSKENNNNKNSRDDRNNSKDRKGGSSMRDRDDMMGRTDDRMDRSDDKKNRNNDRMGSNNDRNGGRNGGRMGGKNNRNDMMGSRDDFFNGREDFPQSDEFGSQDMSQGQYNNYYSTTPSTRRYKRERRTENSGQRSQYNPNTHKITGYEDNFRSDEKNSTEHHSGNETDNKACALHCFLENLEMTAEDGMPDRYLVTHAITKDVKNEDLRDFLQESIEECFQILDNENTEDKCEFSKNLLICLSEKGRANCDDWKDDLKF

>PsauOBP17

MWFRAMVLVAGLAAAHAVAMDEDMAELARMVRESCVDETGADVALVEAVNGGADLMPDDKLKCYIKCTMETAGMMSEGEVDIEAVLALLPPELAAHNAPALRACGTVRGADHCDTAWKTQVCWQNANKADYFLI

>PsauOBP18

MSKFTCLVLCLVAASVSRVYAGEEENKAAFREAIKPIIDECSKEYGVSNDDIDAAKTAGSADAIKPCFLGCVYKKAEVFNAKGEYDVDNALIKLKKFVPDESKFAQFAEIGKKCASVNEKPVSDGEAGCERGSLLTACFLEHKAEIPV

>PsauOBP19

LKGGDYKPRRVNVPAFLVKRYQMECLEETRVDANSIISAIHRRWKFPRGQNTLMKSWALCVLMKNQIMTKEGVYKIDVALKRIPEEDRDIVETQIDKCLTQKAIPAP

>PsauOBP20

MSKFTCLVLFAIAVNFNGVHGFGAVQAIFSGCAKEYGLTEDSLQVIMSAKDVAKLDPCFWACMLKKAEFINDKGEYDPEAGMAYLEKIMPLNKENEMIKEIATNCKSVNDETVSDGEAGCERGALVAVCVMEQVETRKKQMAP

>PsauOBP21

MKRYSKVTEDVEPFRKNLSECARQVKASMADVEHFLKRIPQANMEGKCFVACILKRNLIIKDNKISTNQLLEANRAVYGDDSEVMSRLQMAIGECVKAVDGIFEICEYASVFNDCMHIKMEHVLDQVMMERRLEAVSKMTSDPEQWGDTEDEILKLVKDEL

>PsauOBP22

MIRSCSGLVLVAVFQVIFGQAPQGRPSSSPGFQRQTLRVPEHCLAPPPGVDLHKCCPIPKLFSDEAMDRCGIDKMTKEELGKPSKPKIPCQESYCLMRNANMLRENKSVDYEELRAFIDKWAEVDPDFTVPISSAKMVCAKDGGPSGPPVCEPDRIFICLTSNVLWNCKLQNIDDNGGCKILKEHMDECRPYYWSPSEQVELESRKSQ

>PsauOBP23

MDFWNAQNSLKSHDLGCALVCVFEKNEFLSEDATQLLAANINAFYKASGADDRMSQKMLELIQLCKKSTRTISSLCNKALELAKCFRYGILMMHWAPAPNYWLNKTEFTPGMEPEQVSDDQSLDPTIRSEMNQRRLFATLFRGCRK

>PsauOBP24

MRLLEFKHVIQTYKENFTKTVEVCRNETDYTDEVITEENENDDPYGYGNAGLTNFIYCFLGKTGFINTELFKIDIVLEVTPQENVTRIVEHCKSQIAKKIITYNIPNFSACLVQGLSMYIRSTGVGSYPYGEEQYTTKIVDICHNETGFEKVLTKNFSERKGLREFNDCFLE

>PsauOBP25

MVARIFIIASVACAFINLASGLKEPFPGFKPCLSRDNKCMTQNAKDGLLSFVDGLPEYGVKGSDPDYYDKIDASTNNLKFILKHVTITGLRTCKVNRVERDVDKSTILLELQCDLKLAGDYEMKGRLLIIDLEGKGRIEAELPSMLNTVTCNYVMKTGKDGKEYIRVKNYEHTYKLQKKTTLKFDGLYPHNEVLGKAASDVITKNPDEVVHEIGLEVFKAVIKRLVGHVNNFAGAIPYNELALD

>PsauOBP26

MNSNTSSKSLIYLVINLVHYFQALNEEEKDKIKIKFMTVGATCALEHPIPFEEFATFKSGELPMNGNAGCFTACMFKNLDLFDSKGTWSYENGLKA

>PsauOBP27

MYILSYSKVIVHNETDQVLNIVHDCQEENGFGDTVLQNGFTEENITDEKSLRKFNDCFLEKAGFVSSDGNLNIDAALDKLPPSFAKPIVEHCQAKIILNYTTESVPDFSTCYLDETLNHITMTKKVGYWPFTGAQVLFVPGDSFVDTILTF

>PsauOBP28

MLQFACFVVCAVVVSLSRIHAIEEVAHPEEFMSLVEECAKLHGYTDEQLNKALTLDGVETIKPCFWGCAFTKAGFLNGHGQYDLDSGLTSIKRFLGSDSVRYSRFERIARQCESVKDKAVNDGEAGCERGALAAACFMDQLQPRRV

>PsauOBP29

MSQFACFILCIVIVGTNSAHAEGSDEADSSDEYYMLKEECVKKFEITQDDLDAAVKSGDVSKIDPCYWGCHFKKLGLLNDKGQYNLDNYVTNLKKFLKDKDEIAALEDMGKQCASVKDTAVSDGEAGCEMGALLAACVLVYDEKTE

>PsauOBP30

VIKSQIDNFPLIVDVCRNSTGYTDEVLAEEEKTELSKDSGLKKFNSCFLDKTGFEASDEKFDIDEILGEIPQSNTTEMIEHCQSILSEREIPNNVPNFSACLHLAVSGYLRTSKPYHYA

>BmorGOBP1

MWKLVVVLTVNLLQGALTDVYVMKDVTLGFGQALEQCREESQLTEEKMEEFFHFWNDDFKFEHRELGCAIQCMSRHFNLLTDSSRMHHENTDKFIKSFPNGEILSQKMIDMIHTCEKKFDSEPDHCWRILRVAECFKDACNKSGLAPSMELILAEFIMESEADK

>BmorGOBP2

MFSFLILVFVASVADSVIGTAEVMSHVTAHFGKTLEECREESGLSVDILDEFKHFWSDDFDVVHRELGCAIICMSNKFSLMDDDVRMHHVNMDEYIKGFPNGQVLAEKMVKLIHNCEKQFDTETDDCTRVVKVAACFKKDSRKEGIAPEVAMIEAVIEKY

>BmorPBP1

MSIQGQIALALMVYMAVGSVDASQEVMKNLSLNFGKALDECKKEMTLTDAINEDFYNFWKEGYEIKNRETGCAIMCLSTKLNMLDPEGNLHHGNAMEFAKKHGADETMAQQLIDIVHGCEKSTPANDDKCIWTLGVATCFKAEIHKLNWAPSMDVAVGEILAEV

>BmorPBP2

MKLQVVLVVLTVEMVCGSRDVMTNLSIQFAKPLEACKKEMGLTETVLKDFYNFWIEDYEFTDRNTGCAILCMSKKLELMDGDYNLHHGKAHEFARKHGADETMAKQLVDLIHGCSQSVATMPDECERTLKVAKCFIAEIHKLKWAPDVELLMAEVLNEVSWKS

>BmorPBP3

MARYNIVVAVLVLGVVGARGSSEAMRHIATGFIRVLDECKQELGLTDHILTDMYHFWKLDYSMMTRETGCAIICMSKKLDLIDGDGKLHHGNAQAYALKHGAATEVAAKLVEVIHGCEKLHESIDDQCSRVLEVAKCFRTGVHELHWAPKLDVIVGEVMTEI

>BmorOBP5

MKQRLRVLLLRFCILQTVLSESGVDVVKNLSLSFARFFLECDEERHFQPEVRLKVMTFWYSESSTWDRDVGCAFLCIFKKMEIDNPQDPSYRTHLELLSFANSEDNKIANQMVEIFYACGENTETDPCLWALEQVKCYKNRINQLGLTPTF

>BmorOBP7

AVTEEELKIEFTKLVMKCTKDHPVDMSELMQLQQLIAPKKTESKCLLACAYKLNGVMTSQGLYNLEHAYKIAEMSKNGDEKRLENGKKVADICVKVNDVEVSDGE KGCERAALIFKCTLENAPKVFKFGSSEYNCQ

>BmorOBP8

MLRVVVICVCFLVIAPYGINASSLDDLKMVYKNVIKECVGDYPITAADLKLIKARQIPNDDIKCVFACAYKKTGMMTEEGMLSVEGIKDMSQKYLSDNPEQLRKSKEFAEACSSVNDQQVSDGTKGCERAALIFKCSTEKITNFGFEL

>BmorOBP9

MLRVVVICVCFLVVAPYGINAVSYEQKIKIRDQLDRAGFECFKDHKITEDDIKNLRANKPATGENVPCFIACVMKKTGVMNDQGVIRKGPVLELAKKVLADDKDIKKLQDYIHSCSHVNSETVHDKGKGCEFAMQAYTCMSANASKFGFNI

>BmorOBP10

MLRVVVICVCFLVIAPYGINAVSDEQKIKIREQIDKSGFECFKDHKITEDDIKNLRARKPATGENVPCFIACVMKKTGVMNDQGVIHTEPVLQLAKKVLTDDKDIKKLQDYIHSCS HVNSKTVHDKGQGCEFAIQTYTCMSANASKFGFDV

>BmorOBP11

MSANSFVVLAFCALAVGVNALTEEQKAEITKSSLPLIAECSKEFSVNQGDIDAAKKLGDPSGLNSCFVGCFMKKAGIINASGLFDVAATIEKSKKYLTSEEDLKAFEKLTETCAPENDKPVSDSDKGCERAKLLLDCFVANKGSFSVFSL

>BmorOBP12

MTSFMVFFVLSVLTLKYSDALTDEQKNKIQSKFIEIGAECIVEHPISIDDINSFKNKKFPSGVNAGCFVACIFNKIGLFDDKGNLSHNSALEKAKGIFNADEEVKNLEEFLNRCAKVNGEAVGDGVKGCERAKLAYNCLIENSLEFGFNIDF

>BmorOBP13

MLKIHVLLCFGMAILYFGSAKAVTPEESKAFEAFAKPVIEQCQKDFGMDKESFAQKNLDEIDECLIACVVEKFGITNDEKIDGDALKALVTKFVGNEEERNKINKIVEECTEDANKSGDGTCNTSTILFLCLLKNGKDLWGF

>BmorOBP14

MSRQQLKNSGKMLKKQCMGKNDVTEEEIGDIEKGKFIEQKNVMCYIACIYQMTQIIKNNKISYEASIKQIDLMYPPELKESAKASAGRCKDVSKKYKDICEASYWTAKCMYEDNPKDFIFA

>BmorOBP15

MFLKNIFIECVLLYFVMLNTSFVNTMTKQQIKNSGKILKKACISKNDVTEDQISDIDKGKFIEDKNVMCYIACVYSMSQVVKNNKFVHDAMVKQVDMMFPTEMRDAVKASIANCRGVAKNYKDICEASFWTAKCMYEFDPANFVFA

>BmorOBP16

MRISFLFLISVTIITFDSVFAMTRAQVKKTMTIMKNQCMPKNGVTEDQVGKIEEGIFLENHNVMCYIACVYKTIQVVKNDRLDKDLISKQIDVLYPQEIRESTKKAVGDCINLQEKYDDWCEGIFRSTKCLYEKDPANFIFP

>BmorOBP17

MTRQQLKNSGKIMKKTCMPKNDVTEEEIGQIEQGKFLEQRNVMCYIACIYTVTQVVKNNKLSYDAVIKQVDVMFPAEMRPAVKAAAENCKDISKTFKDICEASYWTAKCMYDFDPKNFVFP

>BmorOBP18

MILIVIAKFLILISLCETMTMKQIKNTGKMMRKSCQPKNNVDDEKINPINDGVFIEENEVKCYIACIMKMANTMKNGKLNFEAAMKQADLLLPDEMKEPTKEAIVACRKVADSYKDVCDASFHVTKCIYNHNPSVFFFP

>BmorOBP19

MTSAKTDVEIKAWFLGQAVECSKDHPVTTEELRMHKHELPDSKNAKCLMKCVFRKCNWLDSKGMYDINAAYASSTKDFSDDKTKQENANKLFDTCKSVNEENVGDGEEGCDRSLLLAKCLTKAAPQVSIYYS

>BmorOBP20

MAVHIFLILASYMALAAHGQLDDEIAELAAMVRENCADESSVDLNLVEKVNAGTDLATITDGKLKCYIKCTMETAGMMSDGVVDVEAVLSLLPDSLKTKNEASLKKCDTQKGSDDCDTAYLTQICWQAANKADYFLI

>BmorOBP21

MITASLHVIFALLAFVYGGKDKPVLSEEIKEIIQTVHDECVGKTGVSEEDITNCESGIFKEDVKLKCYMFCLLEEAGLVNDDGTVDYEMFTSLIPEEYFDRATKMIFSCKELDTPDKDKCERAFEVHKCSYEKDPDFYFLF

>BmorOBP22

MLKVFVVVVCTLGASQLCAALYTQKVAVSFPKDKTTIVVEAMKSCIAKTGANPNVIEVISSGKVSEDEKFKEFFYCACNDIGVVNPDGHIKVKECIELFPKETQPLVEPVI KNCDKEGVNKYDTLFKYLKCFQETSPVRVTLA

>BmorOBP23

MTSKVLLSCVVLAVLATTVLAEDSRKLVSFAPEVAKKLKVLIQECLNENGLGEDAIEVIRAGEYREDEPFQNLVYCAYKKFGALDENNRIISQVAAASFPKDIDVVTVIESCGKEDGNTPVEQVFKYFKCFQKNSPVRMQLY

>BmorOBP25

MKSVVLICLAFAVFNCGADNVHLNEDEREKANWYTAECGVETGVSTEVINAAKIGKYSKDKAFKKFVLCFFKKSAILNSDGTLNMVVALAKLPSGVNKSEAQSVLEQCKNKTGQDAADKAFAILQCFHKGTKTHILF

>BmorOBP26

MKSVVLICLAFAVFNCGADNVHLAETQKEKAKQYTSECVRESGVSTEAINAAKIGKYSKDKAFKNFVLCFFNKSAIFNSDGTLNMDVALAKLPPGVNKSEAQSVLKQCKNKTGQGAADKAFEIFRCYYKGTKTHILF

>BmorOBP27

MKSVVLICLAFAVFNCGADNVHLTETQKEKAKQYTSECVKESGVSTEVINAAKTGQYSEDKAFKKFVLCFFNKSAILNSDGTLNMDVALAKLPPGVNKSEAQSVLEQCKDKTGQDAADKAFEIFQCYYKGTKTHILF

>BmorOBP28

MLKVFIVTFFAFQLSAIARLQANGCVAVPFPKDKTIIIVEAMKSCIAKTGANPNFIDVIRSGKVSEDEKFKEFYYCTCNDTGFVNPDGHIKVKECIELFPKETQPLVEPVIKNCDKEEGVNKYDTLFKFLKCFQETSPVRVALA

>BmorOBP29

MTGPAAAAVLLALLAAAGQATTGCKNCVILGKEERAMFRSHSDACLAQSRVEPRLLESMMNGELIDDAALRKHVYCVLLSCKMIGKDGKLLKAAILGKLAARPAGRDVTKVLEACAEQPGASPEDVAWNIFRCGYNRKAVLFDYMPAGGASSGNTENHP

>BmorOBP30

MRSFVILLNYGLLCCGQFMAEDYYYDIVTRDPDDLMREKENEVRALRAFQADCAEDVQVKPDLVVNLKSGDWQTEDVSLKKWALCVLMKLGLMTAQGVFKMNEAMSKIPDMNDKIIAEKLIDDCLSLQATTPHDAAWNYIKCHHQKDPEGNFSSLNIF

>BmorOBP31

MKTFIVFVVCVVLAQALTDEQKENLKKHRADCLSETKADEQLVNKLKTGDFKTENEPLKKYALCMLIKSQLMTKDGKFKKDVALAKVPNAEDKLKVEKLIDACLANKGNSPHQTAWNYVKCYHEKDPKHALFL

>BmorOBP32

MYSHKYLNDFTNIPEILIILLSSVALMSYGYNTKLFSHSLGSEPSLSILYARDKKSDKVTNECLMEMYPKNLYKYPLRIDRNDIPCIIHCVLKKFGIISNDGFINIKNYYRRVQAIHRYDPRILISDVGETCAQNINGMNLDHDVCKKAKVFNDCTQLYAISYREPEDW

>BmorOBP33

MYAHDKLSDMIADQCLNEMYPRSKRLEIEESDEPCIIFCVLKKFGIMSPTGVINLEAYRKRVQLPEQLAQRNSINDFGSACLESAEATQHKQDVCKKAKVFNECTHLYKILLK

>BmorOBP34

MEKMILLNVFAVVLPCVLASRTRGSSGTLVDFTDPKVQGHLDALVRMAQSCVIKVRATPKDVRAYFTNSSPVSRSGQCFATCMLEQSDIINHGKVNRDLLVHLAGLVNGKNSRVVRKLNSVSRLCLDSISGMTDRCQLASTYNDCLNENMIEFAFPLDIAEEAVRKMPFHLIQPK

>BmorOBP35

GMSTHVLDFKRNMTECLKEVQNNDKRPIKRLSPKQESPIHGECLIACVLKKNGVIQNGKVNKDNLMALVSKFHAKETKLMKKLEKNLDRCINISVKNHDECSLASQLNDCTNDIMASSKQKILFNY

>BmorOBP36

MAVSEISRILTFLTIVSFIYIVYSFKPLTKDEHIERYNKMNEDIEPFRKNLTECARQVKASMADVEKFLKRIPQSNMEGKCFVACILKRNSLIKNNKLSQENLLEVNRAVYGDDSEVMSRLKTAILECSKIVEDIFEICEYASVFNDCMHMKMEHILDKITMERRMEALGQMSSNPDEWSEEEDEMLKLVKDEL

>BmorOBP37

MFYPFRFTLLFYGLFVIYLVRAEPEKENHFTLALKKTLFSTARSCMSHVNANETDLEYLRKDPPFPDKAACIIKCLLEKIGVVKNNKYSKMGFLTAVSPLVFTNKKKLDHYKSVSENCEKEINHDQTTECELGNEVVSCIFKYAPELHFKT

>BmorOBP38

MANLVLLLTFVLMTLSMARLKSTEAPKSKTALFNDQDNMGYEELDMEEIMSACNESFRIEYAYLESLNDSGSFPDETDKTPKCYIRCVLEKTEILSENGVLNPATAALVFAGERNGKPMSDLEEMAVACADRHEKCKCEKAYNFVKCLMYMEIDKYEKKN

>BmorOBP39

MVRKISALLCCFCVLGISMCDSAISTDNEQRCKNPPTAPQKIERVITLCQDEIKLSILREALDVIKEEHTMPAERKRNKREVPFTHDEKRIAGCLLQCVYRKVKAVDGFGFPTLEGLVGLYSDGVNERGYFMAVLEASRECLMKNHDKFSRTTPMDNGRNCDVSFDIFECISDRIGEYCGTSGL

>BmorOBP40

MSEFIQPSWRTQCNFRLNWDNRNRLSIDISHGAATTQTPVPTTKPKALRDFMVVPQSCDKTTCVFKKLNIVSDKGVVDVKSFIKLLDKFTNSYPVWNSAKARVITTCLRKSLIAYDGGCELNNILACTFDVLSENCPLNGNNQTC

>BmorOBP41

MLTILFLLPIVVGVLSGNIPEQPRVYCGELPNTIYSCLGNPKIIQPEVSEKCNKPISECDKTRCIFKESGWAKNNVIDKKKVSDYFEQFAKDNPDWSAAVQNFKTTCLSDSLKPQGVDTNCPAYDIIHCALISFIKFASPSQWSTSEQCVYPRQYAGACPVCPERCFAPSVPNGSCNACLALLRTP

>BmorOBP42

MMGYACVFVILAVLQAISAEDPPGLPPFLKDAPEKCKSPPRVKNPNECCISEPFFKEADFIECGIEKPGSERGPPDCSKQNCLLKKYNLLKNDETPDIEAIKSLLDKYIEKNPSFKSSVEKAKECLREDLPGPPQICLANRMTLCIGTVLLMECPDEKWNTTDDCKAFKDHMTECQKYFPK

>BmorOBP43

MKVCVLFAIFTVAQAAKATLKPISACCNIPELGNPEPLAECSNPKLPGPCKDIQCVFEKSGFLTENKTLIKEAYKTHLRQWAKEHEGWSVAVEKAISDCVDKDLRQYLEFPCSAYDVFTCTGIAMLKKCPNEHWTC

>BmorOBP44

MSRLVLFFTILVVLQEFIINLYFNFITEIDSCCVKKYPKLFDSEFITECYNTQRKANDKCERDMCVARKLNLLTEEDSINKDALLRFVEEGFKTEIDLVNAIKKKCFEEDISNIGKPEMCEVAKYKICITSRMAEDCPKWDSKGICSSAQQKVENFMKMLS

>SlitPBP1

MANARWRFVFVVYALYLTSAVLGSQDLMVKMTKGFTRVVDDCKTELNVGDHIMQDMYNYWREDYQLINRDMGCMLLCMAKKLDLMDDQTMHHGKTEDFAKSHGADDDVAKKLVSVIHECEQQHAGIADDCMRVLEVAKCFRTKIHELKWAPSIEVIMEEVMTAV

>SlitPBP2

MAFCPSVTMSLRVALVVAASLLVVVQASQDVMKNLAVNFAKPLDDCKKEMDLPDSVTTDFYNFWKEGYELTNRQTGCAILCLSSKLEILDQELNLHHGRAQEFAMKHGADEAMAKQIVDMIHTCAQSTPDEAADPCMKALNVAKCFKLKVHELNWAPSVELIVGEVLAEV

>SlitPBP3

MGSRNVFVALVVLTVGMREIEPSKDPMKYIASGFVKVLEECKHELNMNDHLIADLFHYWKLEYTLLNRDTGCAIICMGKKLDLLDASGRMHHGNAQEFAKKHGAGDEVASQIVQIIHDCEKKHERDDDECLRVLEVAKCFRTGIHELNWQPNVEVIVSEVLTEI

>SlitGOBP1

MLLLLRALPLLAAVLPLRADVNVMKDVTLGFGQALDKCRQESQLTEEKMEEFFHFWREDFKFEHRELGCAIQCMSRHFNLLTDTSRMHHENTEQFIQSFPNGEVLARQMVELIHACEKQHDHEEDHCWRILHVAECFKQACVQRGIAPSMEIMITEFIMEAEAR

>SlitGOBP2

MTSKCCLLLVLMAAATSSVMGTAEVMSHVTAHFGKALEECREESGLSAEVLEEFQHFWREDFEVVHRELGCAIICMSNKFSLLQDDSRMHHVNMHDYVKSFPNGHVLSEKLVGLIHNCEKQFDSMTDDCERVVKVAACFKVDAKAAGIAPEVAMIEAVMEKY

>SlitOBP3

MWMQALVLTLATLATLAAAAVEMDEDMAELARMVRDNCAGETGVDVALVEKVNAGAELMPDDKLKCYIKCTMETAGMMADGEVDIEAVLALLPPSLAEHNAPAL RACGTQRGADHCDTAFRTQQCWQNANKADYFLI

>SlitOBP4

MTKVLFAIVLTMITFAVVLSASTKEAMTTTMSDQVNSIDVDVLAVMDMCNDSYRIDPTYLQALNESGSFIDETDKTPKCFIRCVFENVGIVSEDGKQFNPARAAVIFAGERNGKPMEDIADMTALCATDRQETCPCDRSYKFLRCLMSMEIERYEKS

>SlitOBP5

MSVVRCSSLLVAIFCFVSVNAISGDEEAGIKDALRPFVQECADEFGITEEQFEEAKKKASAADIDPCFMSCFLKKAEFFDSQGKFDVDSTMAFAKEHLTSEPAMKFVEAVGD ECVKINDEDVSDGDKGCDRAKLLFECIAETKKKME

>SlitOBP6

MSKFTCLVLCVVAVSLSGVHATAEEKAAFIEAVKPYVQECSKEHGVTPEDIKSAKAAGNADGINSCFLSCVYKKAEVITEKGEYDADKALEKLKKFVSNEDDYAKFANIGKKCASVNEKSVSDGEAGCERAALLTSCFLEHKSEISA

>SlitOBP7

MDQKRICLFVIAMFLASGSDAMSRQQLKNSGKMLKKNCMNKIGVTEDQIGSIDKGKFIEDRKVMCYIACIYELTNVIKNNKLNYEASIKQIDLMYPPDVKESAKAAVEKCKDVQKKYKDICEASFYAAKCMYEFKPEDFIFA

>SlitOBP8

MLLTKIVKFFILVATCEAMTMKQIKNTGKMMRKTCQP KNNAEDEKIDPISDGVFIDEKEVKCYMACIMKMANTIKNGKLNYDAAMKQADLLFPDDIKEPAKEAITACRKVADAHKDICDASFHVTKCIYNHNPGIFYFP

>HarmPBP1

MEFHRSTMMSVRLALVVAVCLFIRVDASQDVIKNLSMNFAKPLEDCKKEMDLPDSVTTDFYNFWKEGYEFTNRQTGCAILCLSSKLELLDQELKLHHGKAQEFAKKHGADDAMAKQLVDLIHGCAQSTPDVADDPCMKTLNVAKCFKAKIHELNWAPSMELVVGEVLAEV

>HarmPBP2

MAASRWLFARAFCLVLMMGSAMSSKELLTKMTGGFTKVVDACKTELSVGDHIMQDMYNFWREEYQLVNRDLGCMIMCMTAKLDLIGDDQKMHHGKAEEFAKSHGADDALAKQLVGLIHGCETQHQAIEDHCSRALEIAKCFRTKIHELKWAPSMEVIMEEIMTAA

>HarmPBP3

MGSRHVFFALVVLAVSVRKAEPSKDAMQYITSGFVKVLEECKHELNLNEQILADLFHFWKLEYSLLGRDTGCAIICMSKKLDLLDANGRMHHGNAAEFAKKHGAGDEVASKIVTIIHECEKKHEQDGDECLRVLEVAKCFRTGIHELNWQPKVEVIVSEVLTEI

>HarmGOBP1

MPGVLRALLVLAAAAPLLADINVMKDVTLGFGQALDKCREESQLTEEKMEEFFHFWRDDFKFEHRELGCAIQCMSRHFNLLTDSSRMHHDNTEKFIQSFPNGEVLARQMVELIHSCEKQFDHEDDHCWRILHVAECFKGSCVQRGIAPSMELMMTEFIMEAEAR

>HarmGOBP2

MTSKSCLLLVAMATLTASVMGTAEVMSHVTAHFGKALEECREESGLSAEVLEEFQHFWREDFEVVHRELGCAIICMSNKFSLLQDDSRMHHVNMHDYVKSFPNGHVLSEKLVELIHNCEKKYDTMTDDCDRVVKVAACFKVDAKAAGIAPEVAMIEAVMEKY

>HarmOBP1

MSKFTFFVLCVVAVSLSKVYASDEDKAKLHEALKPLVEECMKDHEVSLDDLKAAKEAKSADGVKPCFLACVYKKAEVLNDKGEFDADHALEKLKEFVSDEDVLAKVAEVGNTCKAVNDKAVSDGDAGCERAALLTACFLEHKAEILV

>HarmOBP2

MMDRKRLCLLIIAMFLAQGSDAMSRQQLKNSGKMLKKNCMNKNQVTEDQIGSIDKGKFVEDKKVMCYIACIFEMTNVVKNNKLNYDASIKQIDLMYPPDLKESAKAAVEKCKDVQKKYKDICEASYWTAKCMYDFKPEDFIFA

>HarmOBP3

MSKFTCFVLCVLAVSLGEVRSNALEKAAIRAAVYPLIVDCAKEHAVTLEQLKAAKASHSAEGINPCFQSCVYKKTGIFNDNGEYDVANAKTKLQKFVTDEDEYARIAEVGKTCASVNDKSVSDGAAGCERAALLTACFLEHRAQIII

>HarmOBP4

MSKLTCVVFAAVAVVFSNVNADDETRASFRQVLGPLVMECRNEFGITEDDLKKAQQERSPDALKPCFIACVFKKFGIITSAGKYDSDASISRIKDVVKNDDLLAKLKSVGEKCNSVNDASVSDGDAGCERAALLAKCFIENKSELSI

>HarmOBP5

MSKFTCLVLCVVAASLSQAYASEEEKAAFREAIKPIVEECSKEHGVSHDELKSAKDNQNADNIKPCFLGCVYKKAEVFNSKGEYDVDKALEKLKKFVSNDEAYAKFAEVGKKCASVNDKAVSDGDAGCERGALLTACFLEHKAEVPL

>HarmOBP6

MSKFTCLLLCVVAVSLSKVHATEEEKEAIRAAVRPIMQECGKEHGVTLDDLKAAKAAHSADGIKPCFQSCVYKKAGIFNDNGEYDIANAKTKLQKFVTNDEEYARIAEVGKMCASVNDKPVTDGAAGCDRAALLTACFLEHRAQIII

>HarmOBP7

MFRFGVLSFVVLLFCMESSYALSSEEELSIKEALHPFVVECAEEYGMTEEMFEEAKKKGSAEDIDPCFMSCFLKKTGFFDDSGKFDAEKSISFAKEHITSESAIKFLEAGAGECVKINDEDVSDGENGCDRAKLLFDCLTELKKKMSE

>HarmOBP7.2

MSRFGVLSFVVLVFCMENIYALSSEEELSIKEALHPFVVECAEEYGMTEEMFEEAKKKGSAEDIDPCFMSCFLKKTGFFDDAGKFDAEKSISFAKEHITSETAIKFLEAGAGECVKINDEDVSDGDKGCDRAKLLFDCLTDLKKKMSE

>HarmOBP8

MLLIEIVKFLTLVAMCEAMTMKQIRNTGKMMRKSCQPKNNVADEQIDPIAEGVFNEDKEVKCYMACIMKMANTIKNGKLNYEAAIKQADLLLPDDIKEPAKEAITACRKVADAYKDICDASFHITKCIYTQNPGIFYFP

>HarmOBP9

MCKFSVLFLYSAVMAVNIWSASCISEEDKAAIITAIAPLAQNCGSECGLDNDDFEKYKEDGSDMDPCFKACLMTQMGVLDKEGKYDGKGLHKAMEEADYPGDKDDAQKFLDELDRCFDAKGDNSGSDEEAKMKRADVLFRCMQDMKEK

>HarmOBP9.2

MCKCSVVFLYLAVMAINIWRASCLSEEDKAAIITAIAPLAQNCGSECGLDNDDFEKYKEDGSDMDPCFKACLMTQMGVLDKEGKYDGKGLHKAMEEADYPGDKDDAQKFLDELDRCFDAKGDNSGSDEEAKMKRADVLFQCMQDMKEN

>HarmOBP13

MFTGTLPLVVFLATFAYGGKEKPVFSDEIKEIIQTVHDECVAKTGVAEEDITNCENGIFKEDPKLKCYMFCLMEEASLVDDDDAVDYDMLVSLIPEEYVDRTTKMIFSCKHLDTPDKDKCQRAFEVHKCSYEKDPDLYFLF

>HarmOBP18

MKSFVVFCVLVAGAFAANVSLPPKQNEKANQIATECMKESGLKPEVLAEAKKGHISDDEHLKKFTFCFFKKAGIVSEDGKLNTEVALAKLPPGVDKAEAEKLLETCKGKTGKDVTDTVFEIFKCYHHGTKTHILLGF

>HarmOBP16

MFKLCVVLAFIVATCHGGTLERTSSTCGQIPRELTACLDLQPAVSPEIQEKCRRANECERLTCVFREYNLLDGAEVNKERTAAFLDNFVKQYPSWEVAIDVAKTSCLRSSGLKPQGVFLDCPAYDIIQCVFANLVKNALPSQWSSMSQCNHAREFAAACPICPDACFAPLVPIGTCNACSAARRSS

>HarmOBP17

MRAWSVTLVALLGALGAARAVAMDEDMAELARMVRENCAAETGADVALVERVNAGADLMPDDKLKCYIKCTMETAGMMADGEVDIEAVLALLPPELAEHNAPSLRACGTVRGADHCDTAFRTQQCWQNANKADYFLI

>HarmOBP18a

MTRQQLKNSGKLMKKSCMPKNDVTEEEVGDIEKGKFIESRNVMCYVACIYTMTQVVKNNKLSYEAVIKQVDMMFPAEMRDAVKAAATSCKDITKKSKDLCESAYWTAKCMYDYDAENFVFP

>HarmOBP19

ARTEHEIKEWLFREGVACNKDFPITPDEMMMLKDNKLP DSTNAKCLIACIFKKTGMIDSKGMFDPDKSIAMTEKDFADNPEKLATSKKLMEACRGVNEQAVADGEKG

>HarmOBP20

KVFYLLTVLSACYGAVDITKYFKTCNRNAIDVNDCMADAVQKGIAVMINGIDELGIPPIDPYLQKDFRLEYKNNQIAAKLNMKNIQVEGLRAAKVHDARLRADDDKFHLEVDLTSPKVTVHAEYHGEGKFNSLRILAFGEVNTTMTDLVYTWKLDGVPEKNGTETYIRIKEFYMRPDVGSIVTNFKNDNPESRELTDLGTRFANENWRTLYREFLPYAQANWNKIGTKVANKLFLKVPYDQLFPTSS

>HarmOBP21

FQMSRAQVKKTMSLVKNQCMPKNSVTEDQVGKIEEGVFLEDRNVMCYVACIYKNLQVVKNDKLDMSLITKQIDALYPPELKEPVKKAVSLCIHSQDNYNDLCEKVFHASKCLYEKDPASFIFP

>HarmOBP22

MTREQIKNSGKLIKKTCMAKNDLSEDQVKDVDKGKFIEEKPFMCYIACVYKMGQTIKGNTVNHDMMIKQVEMMFPNEMKAPMKAAIEHCRPVVKKYKDVCEVSYWTAKCIYEFDPPNFMFP

>AipsPBP1

MAPHPSVTMYVRLALVIIAGLFITVECSQEIIKNLSLQFAKPLEDCKKEMDLSDTVITDFYNFWKEGYEFTNRQFGCAILCLSSKLELLDQDLKLHHGKAQEFAKKHGADEAMAKQLVDMIHSCTQSTPDVADDPCMKTLNVAKCFVAKIHDLKWAPSMDLIMGEVLAEV

>AipsPBP2

MAASRWCIACLVCVLFAARSVMTSQEVVASFSKGFTNVVEHCKAEVNAGEHIMQDIYNFWREEYQLVNRDLGCMVLCMANKLGLIGEDQKMHHAKAEEFAKSHGADEAVAKQLVAILYECETKHAAVEDECGMALEIAKCFRTKMHELKWAPSMEVAMEEIMTAV

>AipsPBP3

MGTYNVFFAFVLMAAGVREIEPSKDAMKYITSGFVKVLEECKQELNMNDRIIADLFHYWKLDYTLLNRDTGCAIICMSKKLDLLDDTGRMHHGNAQEFALKHGAGEEVASKI VTIIHDCEKKFERDDDECLRVLEVAKCFRTGIHDLDWQPKVEVIVSEVFTDM

>AipsGOBP1

MTQPGQVLVLVLLAAAALADVNVMKDVTLGFGQALDKCRQESDLTEEKMEEFFHFWRDDFKFEHRELGCAIQCMSRHFNLLTDSSRMHHVNTEEFIQSFPNGEVLARQMVALIHGCEKQFDHEDDHCWRILHVAECFKHACVAHGVAPSMEMMMTEFIMEAEAR

>AipsGOBP2

MTLRCCLLLVVVAAVTRSVVGTAEVMSHVTAHFGKALEECRDESGLSAEVLEEFQHFWREDFEVVHRELGCAIICMSNKFSLLQDDSRMHHVNMHDYVKGFPNGEVLSGKLVELIHNCEKQYDTLTDDCDRVVKVAACFKVDAKAAGIAPEVAMIEAVMEKY

>AipsOBP1

MDISKRRSKNAFRRLLVNTWLRLVQIFTCLSAPPVVSADVTSKCQGSKYENECDKLTCVFRKAKWLDGNAVDKAKLITYFEQFEKDHPEWAPAMQNVKTSCLGAELKTQGVFLNCPAYDVMHCVLGSFIKHATPTQWSTSASCSYPRAYAAACPICPEDCFSAQVPFGSCNACYLPPRTP

>AipsOBP2

MSKFTCLVLCVVAASISRVHADDDANKAAFREAFKPILDECSKEHGVSNDDIDAAKKAGSADAIKPCFFGCIYKKAEVFNAKGEYDVDSALSKLKKFVPDEAKFAKYAEIGKKCASVNEKPVTDGDAGCERGAMLTACFLENRAEMLI

>AipsOBP3

MIRSCRCLVFAAVFQVVLGQGLTGTDSGPPGFQRPQSYVPKHCFAPPPGVDLHTCCPIPQLFPDEDMESCGIQKLTKEQYENPSPARIPCQESICLLRNANLLKQNNSIDYEKMGDFVDNWAKMDPDFTIPITNAKKVCLIEGGPPAPPVCEPDRIFTCLTSYVLWNCKLRLDSGEGCKILKEHMDGCRPFLAGP

>AipsOBP4

MFGYQFLSFAAALICFGSSYALTSEEEANIKEAFHPFIMKCAEEYGITEEQFEEAKEKHSAEGIDPCFMSCFMKESGFFDSAGKFDADKTKEFVDAHLTSERAITFMEAVGSECAKVNDEEVTDGDKGCDRAKLMWGCIQDLKEKMEGSE

>AipsOBP5

MKYFVLFVALVAGIHANVTLPPEQSEKALKTASECIKETGVSKEVLAEAKKGHIADDEGLKKFTLCFFKKAGIVDNDGKLNLETALAKLPPGVDKAEAKKVLEGCQAKSGKTPQDTAFEIYKCYHAGAKTHIALAGI

>AipsOBP6

QRENKGASLKPLSVCCDIPELGDPKHLAKCSNPKLPGPCNDVQCVFEESGFLTDKNTLNKEAYRNHLKQWEENNKGWTVAVDKAIKECVDNDPRQHLDIPCKAYDVFTCTGIAMLKKCPDSAWKC

>AipsOBP7

MSKFTCVLCVVALSLSSVYVTRAHKPNLRDAWRSELDECAKEYPVTNDEIDTAVRSGDSSNLNPCFNFCVFNKTGFFTENGEYDLKNGLIKLRKAIRDDEEYTKFEEVATECTEDKNTSCDEKAKCDSANRLSLCFLRFKDKVRI

>AipsOBP8

MYLRSTNGGVRSFPLGESAYTTKIVEICSKETGLKKQVPPEEKEIKFSQRKGLREFNDCYLAKTGVTTSDGKLNIDEALEKLPPGFAKPFVEHCQANIILGYIEENVNDFSTCFHQEVQNHLLSFYGFENYWVMLVLGTSFDKTRFTTLFFDKHFDFWLAERAGFVNL

>AipsOBP9

VFICGVLSLNVKASSLDELKMKYVEMIIECSDTYPITAADTLQLKTKTMPDNESIRCLFACVYKKAGMMNEQGELSVEGVNEMTRRYLSDDPDKIKKSEQFTEACKSVNDVPVSDGTRGCDRAALIFKCTVEKSPDFDLL

>AipsOBP11

MTYKVFILVFLTYVSLATSALAPFITKCKWDDSKCIKESAQKVIPLFADGIPDLHVEKHDPLLIKRVDASSPNLKLIVTDIEVKGLKNCEAKKITRDLKAMKLSVKFLCAVDFKGVYDMKGQLFVLPIEGNGDLTAHVPKIQLNAEVDMVDKTGKDGKKHWGVKSWRHSFELKEKSNVKFENLFPDNEFLRKTTEELIASNGNDVIVEVGPEIIKAVTAKVIESIKKLFDEVPVEELAIDE

>AipsOBP12

MYSGTIFLFSFILLIVSNVTFVSSQMTREQVKNSGKLVKKTCSAKNDLTEDEVKDVDKGKFIEEKKFMCYVACVYKMGQAVKGNSLNHDMMIRQVDMLFPADMKAPVKAAIE HCRPVAKKYKDICEASYWTAKCVYEFDPPNFMFP

>AipsOBP13

MVLIYIVKFLILVAMCEAMTMKQIRNTGKMMRKSCQPKNNVEDEKIDPIAEGIFIDEPEVKCYMACIMKMANTLKNGKLNFDAALKQADLLLPDDIKEPAKEAIIACKKAAEGHKDICDVSFHVTKCIYNQNPGIFYFP

>AipsOBP14

MFDPKTVFYLLTVFSVCFGAVDIRKYLKVCDRNAIDVSDCLTDAVQKGIAVMVNGIEELGVPPIDPYLQKEFRVEYNNNQIAVKMVIKNIYVEGLKDAKVHDARLRADDDKFHLEVDMTSPHVFVKAHYHGEGQFNSLKVVAYGDFNTTMSDLVYTWKLDGVPEKNGSETYVRIKEFYMRPDLSSIVTSFRNENPETRELTELGARFANENWRTLYKEFLPYAQANWNRIGVRIANKLFLKVPYDQLFPSSS

>AipsOBP15

MDHNRLCLLVIAMFLATGSDAMTRQQLKNSGKILKKNCMNKHQVTEDQIGTIEKGKFVEDKKVMCYIACIYELTSVIKNNKLNYESSLRQIDIMYPADLKESAKAAVENCKDVQKKYKDICEASFHTAKCMYDFKPEDFIFA

>AipsOBP16

MFPGSIPFISGCVHLGVSNYFRSTQSNLVVHYEDDQIVDAIYNCQDENGFDEVLSNSTNLEENFPEKEGLKKSNDCFLKKTGFVTSDGKLNIDKTLEKLPPSFVKPIVEHCQANIALNYTTESVENFSSCYHDGILNHIFAATEVGIFPFIQTWKFFVPGTSFADTILILN

>AipsOBP17

MNQLLVFVLIVACVRISNGMTREQVKKTMTVIKKQCMPKNSVTEDQIGKIEQGVFNEDRNVMCYVACVYKSLQVVKNERLDLGLISKQIDALYPPELKEPTKKAVSQCINIQDSYNDLCEAVFHSVKCLYEKDPATFIFP

>AipsOBP18

MKTLFVFAACILLAQALTDEQKEKLKKHRTECLTETKVEEALVNKLKGGDYKTESEPLKKYALCMMTKSELMTKDGKFKKDVALAKVPNAADKPSVEKLIDACLANKGNTPHQTAWNYVKCYHEKDPKHAIFL

>AipsOBP19

MFTGTVPFVLCLVAVAFGGKDKPVFSEEIKEIIQTVHDECVANTGVAEEDITNCENGIFKEDPKLKCYMFCLMEEASLVDDDGTVDYDMLVSLIPDEYYERTTKMIFACKHLDTPDKDKCQRAFEVHRCSYEKDPDLYFLF

>AipsOBP20

MLVINATDYDYEGYGTGNMGEKLLTSVPRPASSSNNINNNDTSRTRRSEPLLNKPDLDQCLSQCVFANLQVVDSRGIPREAELWNKVQSSVTSQQSRSALHDQIRACFQELQSEAEDNGCSYSNKLERCLMLRFSDRKVEGKASTPKPASTEQS

>AipsOBP21

MLKFSVVCLYFSVAAVNFWNVHCISEDEKKAFIEAMKPMVEECGSDCGLTEEDYKKHSKGEDMDPCFKKCMMQKLGFLDEDGKYNRKQLHESISEYTGDKDEAKRVQEQLDSCFDANGDNDGDDEESQMKRVDVLFKCLKEIKE

>AipsOBP22

MSMWFRAMVVVGALAAARCGVVMDEDMAELARMVRESCVDETGADVKLVEAVNGGADLMEDDKLKCYIKCTMETAGMMSDGEVDIEAVMALLPPEMAEHNGPALKSCGTQRGADDCDTAWKTQVCWQNANKAEYFLI

>AipsOBP23

MSKFTYLVLCFVAVSRVYANEDERAAFHEAAKPILVECSKENGVSFDKLKAAKEAGSADGIDPCFFSCVFKKTGVFNSKGDFDLDNSLTKLKEFVSNDEDYAKVAEVGKKCE SVNEKDVSDGEAGCERASLLTACFLEHRAEIPV

>AipsOBP24

MAKLLLAMILTVMTFALTMSATTKDAGTKEAIMTTTVANQDSSIDSNDVDVLAVMNVCNESFRIEMSYIQALNESGSFVDETDKTPKCFIRCVFENVGIVSEDGRMFNPARAAVIFAGERNGKPMDDIADMTALCAADRKETCPCDRSYQFLRCLMSMEIERYEKS

>AipsOBP25

SRKLREAMRPIIEQCSKEHGVTDADIQASKDSNNAASLPDCFNHCLFEKSGFIDKNGRYDRDSGLKNLSKYLKDVNQYNKVVEVTKECASVEEKPATGCELGTRLTACLLDHQTSILI

>AipsOBP26

MSKFTCIVLFVVAASLTKVTQAVSEEEKAVAREAMAPILAECSKAEGVSDEDIEEAKKNPSVDAVNSCFIRCVMRKTDALNEKGLFDSDAALAKIRPFVKSDEDFAKFEEIGKACMSVNDKEVSDGEAGCDRAKLLLACFLEHKAEMLY

>AipsOBP27

DSAISADAESRCRNPPTAPQKIERVITLCQDEIKLSILREALDVIKEEHTMPAQRRRDKREVPFTHDEKRIAGCLLQCVYRKVKAVDGYGFPTLEGLVGLYSDGVNERGYFMAVLEASRECLMKNHDKFSRTMPMDNGRNCDVSFDIFECISDRIGEYCGTSGL

>AipsOBP28

MTHIFSSFIPYMITVSMFSFPVSVKIISPTVPVTVVSSSVIITFMFDMYRLVILSIVAVTTVVADTDLQECRRLVHPHSMRCCKKSADAKEKMMKNDDLKECFDLPKDPVKCEHELCMAKKKGITTSDDKLDKAKFEEVVTKDIDDKDLVADIKANCINGDLTKYGPPDFCDFVKMRHCMSMQILNHCTEWNDFGDCPQLKSIIGDCVKLVAA

>SlitOBP9

MCLVKYHVLVLCVILVGSYALNCRSSGGPKEAELKNIYKKCLKMQEGKNSSKGNSAQDWKEPRVQIQRNDWDRGRVGSKENKNSRDDSRSGSKDKKGDSGMRDNRNDMMSRRDDMMSRGDERNDNRKHRTDDRMGNDNDRSGNRGRGNKNNRNDMNGGRDDRFGRDDYFNGREDFPQSDEYGGDMGQYNNNYYSTTQSSRRYKRERRPSNSGQRSQYNPNNHKISGYEDNFRSDERNTTDNNSSKETDNKSCALHCFLENLEMTGEDGMPDRYLVTHAITKDVKNEDLRDFLQESIEECFQILDNENTEDKCEFSKNLLICLSEKGRANCDDWKDDLTF

>SlitOBP10

MVRKISGLLCCLCVFGISFSDSAISADSESRCRNPPTAPQKIERVITLCQDEIKLSILREALDVIKEEHTMPAQRRRDKREVPFTHDEKRIAGCLLQCVYRKVKAVDGYGFPTLEGLVGLYSDGVNERGYFMAVLEASRECLMKNHDKFSRTVPMDNGRNCDISFDIFECISDRIGEYCGTSGL

>SlitOBP11

MKSFVVFCIVFVVGVCATEKGNKIASECIKESGVKSDVLAEAKKGNLGDDPAFKEFTYCFFKKVGIVGEDGKLNRDVAIAKLPSGVDKAEAEKLLDSCKSKTGKDAVETVYEIFKCYQHGTKSHIMFAS

>SlitOBP12

MKTLFVFAACILLAQALTDEQKEKLKKHRTECLTETKVDEELVNKLKGGDYKMDNEALKKYALCMMMKSELMTKDGKFKKDVALAKVPNPADKPTVEKLIDACLANKGNTPHQTAWNYVKCYHEKDPKHAIFL

>SlitOBP13

MITSCLLVLSAVVQVLLAKQPVFESGPPEPWGPPERTSHPGQFQPRVPKRCWVPPQRINVYNCCPIPTLYPDEDMQSCGFEKLSENKPQKPVYRPEGTCKEGYCVMGKFDLLLANNSVDYVKFREYLDNWAESYPEFANAIHIAKEECAQDGGPEVPPICEPDKLFLCLTSTIFWNCKLRDGEGCAALQEHMNECKQYYTRVMAPTIKDFEVR

>SlitOBP15

MYSINCFIFSVILIVMFDNCFVYSMTREQIKNSGKLIKKTCSAKNDLTEDEVKDVDKGKFIEKKDFMCYIACVYKMGQSVKGSTINHDMMLRQVDMMFPNDMKAPVKSAIEHCRPVAKNYKDLCEASYWTAKCIYDFDPANFMFP

>SlitOBP16

MYRFVILSIVLVSALADDIDIRECGRIFHPPPHGCCKANNAVKNKDMLAEELKDCFDGSGPKDPMKCEIDLCIAKKKGFATDDGKLDIKKFEEVITKEVGSDKDLLDEIKTNCINGDLNNYGPPEFCDFMKIKHCVTLHMMNHCSEWSDDGNCKVVKELVGKCAKVI

>SlitOBP17

MKTFRLLCCILSIFLFFDQSYGMTRQQLKNSGKLMKKSCMPKNDVTEDEVGDIEKGKFIETRNVMCYIACVYTMSQVVKNNKLSYEAVIKQVDVMFPAEMRDAVKAAATHCKETTKKYKDLCESSYWTAKCMYDYDAQNFVFP

>SlitOBP18

MFKLCVFLALGFVACHGAPNSSPGTPNANPGTYCGVTPDNIYRCLNNPRVVTPEVSTKCGSQFTECEKMTCIFRELKWSKRGAIDKAKVRAYFDQYETEHPEWAQAVQHVKAFCLASELRAQGVFLNCPAYDIMQCVLASFIKHASPSVWSTATDCAYPKAYAADCPVCPSDCYSPQIPFGSCNACYTQPRTV

>SlitOBP19

MFRRTLLLFSIIYISACNGQTEAPEKNRMMGIDAVHDNNVKIDKDTIITRNLKLEKRSRGPKSVSNKNEDQIEPDWSYANFPKEVSEHVEKFKKNMTECLKEVQTSDKRPVKRLSPKMESPVHGECLIACVLKRNGVIINGKVNKDNLIALVSKFYSKDTRLMKKLEKNLDRCIEMSVRAQDDCALALVLNDCTNDLMASNKHKIMVNY

>SlitOBP20

MEKILIFTFITLSGFAHARISVMYAHDKLSDLVAQQCLSEMYPKNKRIEIQESDEPCIIFCVLKKFGIISASGVINLDIYRKRVQIAHQLDQKTSIMDYGGSCMENAEATQHKQDVCKKAKVFNDCTHLYRILLM

>SlitOBP21

MARRQRGAMFTEALPLFVILVAVTHGGKNKPVFSDEIKEIIQTVHDECVAKTGVAEEDITNCENGIFKEDAKLKCYMFCLLEEASLVDDDDTVDYDMLVSLIPDEYYERTTKMIFACKHLDTPDKDRCQRAFEVHKCSYEKDPDLYFLF

>SlitOBP22

MSKFTCIILCVVAASLTKVSHAAVTEEEKEAFREAMAPIIAECSEEHGVSEADIKAAKESASADNIKPCFLGCVMKKIEVLDAKGLYDAETGLGKLRKFVKDDDEFAKFEDIAKKCLKVNDESVSDGEAGCDRAKLVLGCFIEHKVEMPF

>SlitOBP23

MAKFSCLVLCVVAASLGSIHVASGESLRESLRPVIVACSQEHGVTDAEIQAAKDAGSPASIKPCFIACVFKKAGFINEQGQLDLETGLKNLRQFVKDDEQYKKLEEVAKKCSQVKDKAVSDGAAGCERGVLLAGCFLEHKTSIII

>SlitOBP25

MAKVTCIVLFVVGVSLSSIQADDGKNESEVEIDVNQIIDDCIEEYHIPRRLFLAAAETGSTHALTPCFWSCCFKGVGVLNSEGQYDIDATLDLSKKIFTDHEYEKVEIIVKKCESVNGAPVSNGNIECEKSVLLADCLFDNAKKHFPNMFGVDY

>SlitOBP27

MYKFVILCSIFVAASNADVAQTLTKRETKASLKPLSVCCDIPELADEFQLAKCSPRPPGPCEDVQCIFEVSGFLTDRNTLNKAAYRSHLQKWEKNHPGWTDSIYKAITDCVDNDPRQHLEVPCKAYDVFTCTGIAMLKKCPDTAWKC

>SlitOBP28

MIVRFLLCLYIVEFYGAHARTDQEIKAWFFREGMDCNIEHPISPKEMLELKENKIPDTNNAKCFVACVFKKTGMLDSKGMFDAENSIAMTQKDFANDPNRLESSKKLLEACKKVNDEAVSDGEKGCERSVLLHKCFVETAPQLGIKLP

>SlitOBP29

MWNLLVVFLAICSCVYARRRSSGAEINGLTEEELKMEFTKLIMKCNKDGEVDMTELVQLQNYVVPTKQSTKCVLACAYKAAEVMNAKGEYDIDHAYKVAEMMKNGDEKRLVNAKKMADLCVKVNELSVSDGEKGCDRAAMIFKCTVENAPKFGFKL

>SlitOBP33

MTCSQALALLALVAISQQATTGCKNCIMLGKEEKAMFRAHSDACVAASRVEPRLVDAMLAGELLDEPALRKHVYCVLLKCKLISKDGKLQKAAVLGKMAARPDAKNATKVLESCADQTGDTPEDLAWNLFRCGYDKKALLFDYMPTNVASETDNNS
