# Supplementary material for: Antennal transcriptome analysis of odorant-binding proteins and characterization of GOBP2 in the variegated cutworm Peridroma saucia
Source: Front Physiol. 2023 Aug 10;14:1241324. doi: 10.3389/fphys.2023.1241324 (PMC10450149; doi:10.3389/fphys.2023.1241324)
Supplement: Supplementary file 1 [file DataSheet1.zip › Data Sheet 1/Supplementary materials/Table S2 (primers).docx]

**Table S2**. Nucleotide primers for gene cloning, RT-qPCR, and prokaryotic expression.

| **Primer name** | **Sequence (5′-3′)** |
| --- | --- |
| **Gene cloning** |  |
| PsauGOBP2-F | ATGACGTCCAAGTGTTGTTTATTG |
| PsauGOBP2-R | TCAGTACTTCTCCATGACGGCTTC |
| **RT-qPCR** |  |
| PsauGOBP2-F | TCGGCTGTGCCATCATCT |
| PsauGOBP2-R | CAGGGTGTCGTACTGCTTCT |
| PsauActin-F | TCATCACCATCGGAAACGAAC |
| PsauActin-R | GCGTACAAGTCCTTACGGAT |
| **Prokaryotic expression** |  |
| PsauGOBP2-F | CC*CATATG*ACTGCCGAGGTGATGAGCC |
| PsauGOBP2-R | AT*GAATTC*TCAGTACTTCTCCATGACGG |

F: forward primer; R: reverse primer; PsauActin: β-actin of *P*. *saucia.* Underlined indicated the NdeⅠ (CATATG) and EcoRⅠ (GAATTC) restriction enzyme sites.
