# Supplementary material for: Antennal transcriptome analysis of odorant-binding proteins and characterization of GOBP2 in the variegated cutworm Peridroma saucia
Source: Front Physiol. 2023 Aug 10;14:1241324. doi: 10.3389/fphys.2023.1241324 (PMC10450149; doi:10.3389/fphys.2023.1241324)
Supplement: Supplementary file 1 [file DataSheet1.zip › Data Sheet 1/Supplementary materials/Table S3 (odorants).docx]

Table S3. CAS number, purity, and resource of odorants for fluorescence binding assays.

| **Odorant** | **CAS *** | **Purity (%)** | **Company** |
| --- | --- | --- | --- |
| ***P*. *saucia* sex pheromones** |  |  |  |
| *Z*11-16: Ac | 34010-21-4 | ≥91 | J&K Scientific |
| *Z*9-14: Ac | 16725-53-4 | ≥93 | J&K Scientific |
| **Host plant volatiles** |  |  |  |
| **GLV*** |  |  |  |
| (*Z*)-3-hexenyl acetate | 3681-71-8 | ≥99 | J&K Scientific |
| (*E*)-2-hexenyl acetate | 2497-18-9 | ≥97 | TCI |
| (Z)-3-hexen-1-ol | 928-96-1 | ≥98 | J&K Scientific |
| (*E*)-2-hexen-1-ol | 928-95-0 | ≥96 | J&K Scientific |
| (*E*)-2-hexenal | 6728-26-3 | ≥97 | J&K Scientific |
| **Aliphatic** |  |  |  |
| Octanal | 124-13-0 | ≥98 | TCI |
| Heptanol | 111-70-6 | ≥98 | TCI |
| Dodecanol | 112-53-8 | ≥99 | TCI |
| Decanal | 112-31-2 | ≥97 | TCI |
| Nonanal | 124-19-6 | ≥95 | TCI |
| (*Z*)-jasmone | 488-10-8 | ≥93.5 | J&K Scientific |
| Jasmonic acid | 77026-92-7 | ≥90 | Sigma-Aldrich |
| Methyl jasmonate | 39924-52-2 | ≥95 | Fluorochem |
| **Terpenoid** |  |  |  |
| Citral | 5392-40-5 | ≥96 | TCI |
| Farnesol | 4602-84-0 | ≥96 | J&K Scientific |
| Linalool | 78-70-6 | ≥98 | J&K Scientific |
| β-myrcene | 123-35-3 | ≥90 | TRC |
| β-pinene | 127-91-3 | ≥95 | J&K Scientific |
| *D*-Limonene | 5989-27-5 | ≥95 | Fluorochem |
| (*E*)-β-farnesene | 18794-84-8 | ≥95 | Macklin |
| β-ocimene | 13877-91-3 | ≥90 | TRC |
| (*E*)-caryophyllene | 87-44-5 | ≥90 | TCI |
| **Aromatic** |  |  |  |
| Benzaldehyde | 100-52-7 | ≥98 | J&K Scientific |
| Indole | 120-72-9 | ≥99 | J&K Scientific |
| Methyl salicylate | 119-36-8 | ≥99 | J&K Scientific |
| Phenylethyl acetate | 103-45-7 | ≥98 | TCI |

CAS^*^: chemical abstracts service number. GLV*: green leaf volatile.
