# Supplementary material for: Antennal transcriptome analysis of odorant-binding proteins and characterization of GOBP2 in the variegated cutworm Peridroma saucia
Source: Front Physiol. 2023 Aug 10;14:1241324. doi: 10.3389/fphys.2023.1241324 (PMC10450149; doi:10.3389/fphys.2023.1241324)
Supplement: Supplementary file 1 [file DataSheet1.zip › Data Sheet 1/Supplementary materials/Table S4 (assembly).docx]

**Table S4**. Summary of the transcriptome assembly of *P. saucia* antennae.

| **Type** | **Transcripts** | **Unigene** |
| --- | --- | --- |
| Total number | 277,514 | 151,541 |
| Total sequence base | 262,617,487 | 103,561,382 |
| Percent GC | 39.43 | 39.03 |
| N_50_ length | 1743 | 1158 |
| N_90_ length | 349 | 269 |
| Max length | 18,712 | 18,712 |
| Min length | 182 | 201 |
| Mean length | 946.32 | 683.39 |
