# Supplementary material for: Antennal transcriptome analysis of odorant-binding proteins and characterization of GOBP2 in the variegated cutworm Peridroma saucia
Source: Front Physiol. 2023 Aug 10;14:1241324. doi: 10.3389/fphys.2023.1241324 (PMC10450149; doi:10.3389/fphys.2023.1241324)
Supplement: Supplementary file 1 [file DataSheet1.zip › Data Sheet 1/Supplementary materials/Table S6 (binding).docx]

Table S6. Binding abilities of recombinant PsauGOBP2 to selected odorants.

| **Ligands** | **Max Conc** | **Fluorescence at max (%)** | **IC_50_ (μM)** | **K_i_ (μM)** |
| --- | --- | --- | --- | --- |
| **Sex pheromones** |  |  |  |  |
| *Z*11-16: Ac | 10 | 41 ± 4 | 6.5 ± 1.2 | 4.2 ± 0.8 |
| *Z*9-14: Ac | 10 | 45 ± 3 | 7.5 ± 0.9 | 4.9 ± 0.6 |
| **Host plant volatiles** |  |  |  |  |
| **Green leaf volatiles** |  |  |  |  |
| (*Z*)-3-Hexenyl acetate | 20 | 86 ± 2 | >30 | − |
| (*E*)-2-Hexenyl acetate | 20 | 69 ± 3 | >30 | − |
| (Z)-3-Hexen-1-ol | 20 | 72 ± 3 | >30 | − |
| (*E*)-2-Hexen-1-ol | 20 | 69 ± 7 | >30 | − |
| (*E*)-2-Hexenal | 20 | 77 ± 3 | >30 | − |
| **Aliphatic** |  |  |  |  |
| Octanal | 20 | 75 ± 2 | >30 | − |
| Heptanol | 20 | 80 ± 2 | >30 | − |
| Dodecanol | 20 | 49 ± 3 | 19.5 ± 0.6 | 13.0 ± 0.4 |
| Decanal | 20 | 73 ± 7 | >30 | − |
| Nonanal | 20 | 85 ± 2 | >30 | − |
| (*Z*)-jasmone | 20 | 75 ± 3 | >30 | − |
| Jasmonic acid | 20 | 73 ± 5 | >30 | − |
| Methyl jasmonate | 20 | 70 ± 7 | >30 | − |
| **Terpenoid** |  |  |  |  |
| Citral | 20 | 76 ± 5 | >30 | − |
| Farnesol | 20 | 100 ± 3 | >30 | − |
| Linalool | 20 | 74 ± 6 | >30 | − |
| β-Myrcene | 20 | 45 ± 1.5 | 12 ± 0.5 | 8.0 ± 0.3 |
| β-Pinene | 20 | 79 ± 9 | >30 | − |
| *D*-Limonene | 20 | 85 ± 5 | >30 | − |
| (*E*)-β-farnesene | 20 | 75 ± 7 | >30 | − |
| β-Ocimene | 20 | 90 ± 4 | >30 | − |
| (*E*)-Caryophyllene | 20 | 90 ± 3 | >30 | − |
| **Aromatic** |  |  |  |  |
| Benzaldehyde | 20 | 74 ± 3 | >30 | − |
| Indole | 20 | 100 ± 8 | >30 | − |
| Methyl salicylate | 20 | 79 ± 2 | >30 | − |
| Phenylethyl acetate | 20 | 39 ± 3 | 10.5 ± 0.5 | 6.3 ± 0.3 |

We considered that PsauGOBP2 had no binding affinities with the compounds if the IC_50_ values > 30 μM and the K_i_ values are represented as “−”. Data are means ± SE, n = 3. Max Conc: maximum concentration. IC_50_^*^: the concentration of ligands halving the initial fluorescence value of 1-NPN; K_i_^*^: the calculated dissociation constants.
