# Supplementary figures and images for: Antennal transcriptome analysis of odorant-binding proteins and characterization of GOBP2 in the variegated cutworm Peridroma saucia
Source: Front Physiol. 2023 Aug 10;14:1241324. doi: 10.3389/fphys.2023.1241324 (PMC10450149; doi:10.3389/fphys.2023.1241324)

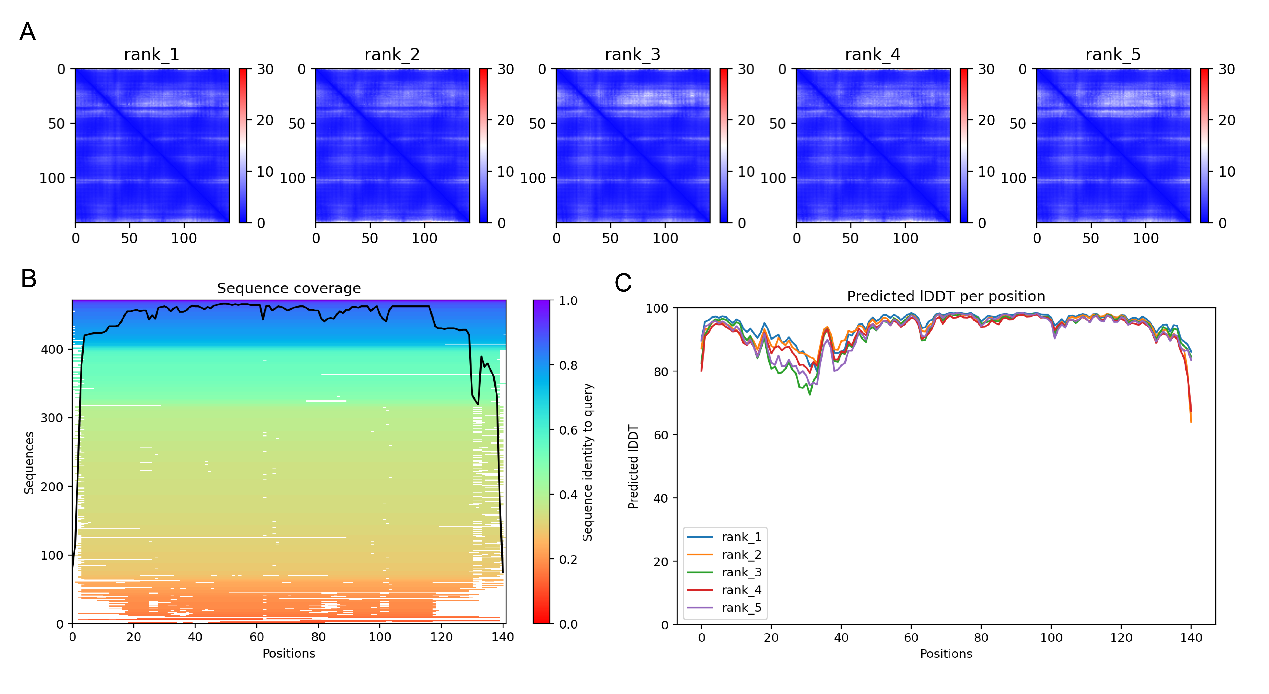

Supplement: Supplementary file 1 [file DataSheet1.zip › Data Sheet 1/Supplementary materials/Fig S1 (pLDDT).tif]
